# How Anterior Crossbite Severity Relates to Appearance-Based Bullying in School-Age Children: Evidence from the ROMA Index

**DOI:** 10.12688/f1000research.179073.1

**Published:** 2026-04-19

**Authors:** Farah M. Babakurd, Khaled Omar, Mayssoon Dashash

**Affiliations:** 1 Pediatric Dentistry Department, Faculty of Dentistry, Damascus University, Damascus, Syria; 2 Faculty of Informatics Engineering, Damascus University, Damascus, Syria; 3 Pediatric Dentistry Department, Faculty of Dentistry, Damascus University, Damascus, Syria

**Keywords:** Anterior crossbite, ROMA Index, dental appearance-related bullying, Olweus Bully/Victim Scale, Mixed dentition, Children.

## Abstract

**Introduction:**

This study examined the prevalence of anterior crossbite in school-age children, investigated the frequency of appearance- related bullying, and determined whether crossbite severity correlates with bullying exposure among children aged 8–12 years.

**Materials and Methods:**

A cross-sectional study involved 2,080 children from public schools in Damascus, using random cluster sampling.

Anterior crossbite and other occlusal issues were assessed using the ROMA Index. Dental appearance-related bullying was evaluated using a modified Olweus Bully/Victim Questionnaire; children reporting bullying two or more times monthly were classified as victims.

Bullying types—teasing, name-calling, social exclusion, and physical aggression—were documented along with occurrence settings. Severity scores were calculated by summing numerical codes for each bullying type. Relationships between occlusal characteristics and bullying were analyzed using Chi-square tests, Cramer’s V, and logistic regression, adjusting for age and gender.

**Results:**

Of 2,080 children aged 8 to 12 years, 19.6% had anterior crossbite, and 34.4%reported dental appearance-related bullying. Children with anterior crossbite were significantly more likely to experience bullying (p < 0.001), with bullying intensity increasing proportionally to crossbite severity. Teasing and mocking were the most prevalent forms, primarily occurring in playground settings.

**Conclusion:**

Anterior crossbite severity functions as a meaningful risk indicator for psychosocial bullying in children, extending beyond simple dental concerns. Incorporating crossbite severity screening into school-based prevention programs offers a practical strategy to improve both oral health and psychological well-being in childhood.

**Abbreviations**
ROMA index: Risk of Malocclusion Assessment index
OHRQoL: oral health–related quality of life

## Introduction

Malocclusion is among the most prevalent oral health conditions globally, affecting approximately 56% of populations. Prevalence varies considerably across populations and diagnostic criteria. ^1^ Beyond functional consequences, malocclusion carries a significant psychosocial burden, particularly during childhood—a period of increased sensitivity to peer judgments and appearance-based evaluation. ^2– 4^

A meta-analysis of 40 cross-sectional studies demonstrated significantly reduced oral health–related quality of life (OHRQoL) in children with malocclusion. Children with malocclusion were 1.74 times more likely to experience OHRQoL impairment than unaffected peers. ^5^

Severity showed a dose-response relationship: Children with normal or mild malocclusion were 56% less likely to experience quality-of-life impairments than those with severe malocclusion. ^6^

Despite robust evidence linking malocclusion to compromised quality of life, the association between malocclusion and school bullying remains contested. A recent systematic review found that 88% of studies reported a positive correlation between dentofacial anomalies and victimization. ^7^

However, a subsequent meta-analysis yielded inconclusive results, reflecting heterogeneity across studies. ^8^

Bullying, defined as recurrent aggressive behavior within a power- imbalanced relationship, carries serious psychological and academic consequences, including reduced self-esteem, academic decline, and heightened risk of anxiety and depression. ^9^

Appearance- related bullying specifically affects 7% to 47.8% of children globally, ^10, 11^ underscoring the social significance of visible dentofacial characteristics in school settings. ^12^

The mixed dentition stage (8–12 years) represents a critical window during which peer awareness intensifies, magnifying psychosocial impacts of visible dentofacial anomalies such as anterior crossbite. ^3^

This sagittal malocclusion occurs in approximately 11% of populations, ^1^ and can compromise facial profile perception, cause functional mandibular displacement and abnormal incisal wear, and worsen skeletal Class III patterns if untreated. ^13^

Beyond functional consequences, anterior crossbite affects dental aesthetics during speech and smiling; its psychosocial effects correlate with severity. ^14^ However, limited research examines the relationship between anterior crossbite severity and appearance-related bullying, especially in Middle Eastern populations. Understanding these psychosocial dimensions is important for timely orthodontic referral and early intervention.

The Risk of Malocclusion Assessment (ROMA) Index provides an objective and standardized, reproducible classification of occlusal disorders, including anterior crossbite. ^15^ The modified Olweus Bully/Victim Questionnaire enables systematic evaluation of bullying exposure, appearance- related victimization, severity, patterns, and contexts. ^16, 17^

Combining objective clinical severity assessment with validated psychosocial measurement minimizes subjective bias and strengthens methodological rigor.

This study addressed a significant knowledge gap: the lack of severity-based analyses of anterior crossbite and bullying, and the absence of data from Syrian populations. The study aimed to ascertain the prevalence of anterior crossbite in children aged 8–12 years using the ROMA Index, estimate the prevalence of appearance-related bullying using the modified Olweus questionnaire, analyze the association between anterior crossbite severity and bullying exposure while controlling for age and gender, and describe bullying patterns and contexts.

The findings are expected to deepen the understanding of the psychosocial dimensions of anterior crossbite during mixed dentition, inform early orthodontic intervention decisions integrating functional and psychological considerarions and support integration of orthodontic care into school-based bullying prevention programs.

## Materials and methods

This cross-sectional analytical study received ethical approval from the Research Ethics Committee at the Faculty of Dentistry, University of Damascus (IRB No. UDDS 2614_28042025/SRC_2320) and authorization from the Ministry of Education (No. 4/443, 02/07/2025).

The study followed the Declaration of Helsinki (2013), ^18^ and written informed consent was obtained from parents or legal guardians of all participating children.

Data collection occurred between July and December 2025, and the research complies with the STROBE guidelines for observational studies. ^19^

### Sampling and study population

A cluster random sampling design was employed across public schools in Damascus, with schools designated as clusters. Children were assessed in private rooms within each school under the teacher’s supervision to ensure privacy and accuracy. ^20^

According to the Directorate of Education, the first cycle included 56,772 boys and 53,761 girls, totaling 110,533 children. Sample size calculation for determining anterior crossbite prevalence (±5% precision, and of 95% confidence level) used the reported global prevalence of anterior crossbite (11%) ^1^ and the standard formula for cross-sectional studies ^21^:

$$n=(Z^{2*}\;P^{*}\;(1-P))/D^{2}$$

With a 95% confidence level (Z) and a 2% margin of error (D), this yielded an initial estimate of approximately 150 children.

To assess the association between anterior crossbite and dental appearance-related bullying, global prevalence rates range from 7% to 47.8%. ^10, 11^ However, no published data exist for children with anterior crossbite in Asia or Syria. Conservative estimates were therefore adopted (P1 ≈ 30% for affected children, P2 ≈ 15% for unaffected) for binary outcome analysis using Chi-square or Odds Ratio methods, maintaining 80% power and 95% confidence level. ^22^ This calculation provided an initial estimate of approximately 236 children. Accounting for a potential 10–15% non-response rate of, ^23^ the sample size was adjusted to approximately 270 children.

Given that the study aimed to estimate the prevalence of anterior crossbite, and the prevalence of bullying, analyze associations within subgroups (such as age, gender, and crossbite severity), and perform logistic regression analyses, the final sample was expanded to 2,080 children to ensure adequate statistical power. ^24^

### Clinical assessment of malocclusion and other occlusal features

A single certified examiner (FB) supported by two school health department staff members performed all clinical examinations. To evaluate intra-examiner reliability, 10% of participants underwent repeat examinations, spaced by 20 to 30-minutes. ^25^ Cohen’s kappa values ranged from 0.85 to 0.95, indicating excellent agreement. ^26^

Occlusion characteristics were classified using the ROMA Index, a standardized grading system that categorizes malocclusions by severity and orthodontic treatment need. The index defines three grades ^15^:

Grade 2 N: Mild malocclusion requiring routine monitoring.

Grade 3 N: Moderate malocclusion with clear need for orthodontic treatment.

Grade 4 N: Severe malocclusion requiring urgent or immediate orthodontic intervention.

Four key occlusal traits were evaluated and graded according to clinical severity and treatment urgency:

• Anterior crossbite: one or more maxillary teeth occluding lingually to the corresponding mandibular teeth in centric occlusion.
• Anterior open bite: Absence of vertical overlap between opposing anterior teeth.
• Deep bite: Excessive vertical overlap of anterior teeth.
• Increased overjet: Horizontal protrusion of maxillary incisors.

All examinations followed standardized clinical protocols. When precise measurements were required to support. ^15^

A calibrated Williams periodontal probe (1–10 mm increments) was used to ensure consistent and reproducible measurements. ^27^

### Assessment of dental appearance-related bullying

Researchers adapted the Olweus Bully/Victim Questionnaire to measure bullying related to dental appearance. ^16, 17^

The modified instrument evaluated the frequency and types of bullying incidents over a two-month reference period. Children were classified as victims if they experienced two or more bullying incidents per month, consistent with prior research. ^28^

The questionnaire assessed multiple forms of dental appearance-related bullying: teasing, name-calling, social exclusion, and physical aggression. Each response was coded numerically (Yes = 1, No = 2) to generate a bullying severity score ranging from 4 to 8 for each participant, with lower scores indicating greater bullying exposure. Researchers also recorded where bullying occurred (classroom, schoolyard, street, or multiple locations).

Before the main study with 2,080 children, a pilot study with 100 randomly selected participants tested the validity and reliability of the questionnaire, approximately 5% of the main sample. The pilot phase evaluated item clarity, validity, and reliability of the modified instrument. ^29^

Results demonstrated robust psychometric properties. Face validity was confirmed by 90% of expert reviewers. The Content Validity Index (CVI) was 0.92, ^30^ internal consistency (Cronbach’s alpha) was 0.85, ^31^ and test-retest reliability measured at two weeks yielded an Intraclass Correlation Coefficient (ICC) of 0.88. ^32, 33^ These findings confirmed the questionnaire’s reliability and stability of use in the main study. Refinements were made to items as needed ensuring the modified instrument ^34^ is aligned with international research standards.

### Inclusion and exclusion criteria

Children aged 8 to 12 years enrolled in public schools were eligible for participation if they could complete both the questionnaire and clinical examination and had obtained parental consent.

The study excluded individuals with chronic medical conditions or disabilities, prior orthodontic treatment, prolonged school absences, or learning difficulties that could affect their ability to respond reliably to the questionnaire.

### Statistical analysis

Data analysis was performed using SPSS software. Descriptive statistics summarized demographic characteristics, the prevalence of anterior crossbite, and the frequency of dental appearance-related bullying. Categorical variables were reported as frequencies and percentages, while continuous variables were presented as means and standard deviations.

Chi-square tests were examined associations between occlusal status and bullying, with Cramer’s V utilized to measure the strength of these associations. Post hoc pairwise comparisons identified specific differences between groups. Ordinal logistic regression assessed how anterior crossbite severity influenced bullying severity, while binary logistic regression determined the odds of bullying victimization across different occlusal groups after adjusting for age and gender. The locations and patterns of bullying incidents were analyzed descriptively using Chi-square and Cramer’s V comparisons. All statistical tests were two-tailed with a significance level of p < 0.05.

## Results

A total of 2,080 children aged 8–12 years were included.

Among the participants, 408 children (19.6%) presented with an anterior crossbite. Of these cases, 15% required immediate intervention, 2.6% showed signs of malocclusion that could persist or worsen, and2% required routine occlusal monitoring. Findings are presented in Table 1.

**Table 1.** Prevalence of anterior crossbite among children aged 8–12 years, categorized by severity and required intervention according to the ROMA Index.

Anterior Crossbite	N(%)	ROMA Grade	N(%)
**Present**	408 (19.6%)	**4n: Crossbite > 2 mm**	312 (15.0%)
		**3n: Crossbite > 1 mm**	55 (2.6%)
		**2n: Crossbite < 1 mm**	41 (2.0%)
**Absent**	167 (80.4%)		
**Total**	208 (100.0%)		

The modified Olweus Bully/Victim Questionnaire identified 34.4% of the children as victims of dental appearance- related bullying. Victimization was defined in accordance with established Olweus criteria, whereby children who reported experiencing bullying two or more times per month were classified as victims. In contrast, those who reported no instances of bullying or only occasional occurrences were categorized as non-victims (see Table 2).

**Table 2.** Prevalence of dental appearance-related school bullying among children aged 8 to 12 years with mixed dentition.

Modified Olweus Bully/Victim Questionnaire	N(%)
**Victims**	715 (34.4%)
**Non-victims **	1365 (65.6%)
**Total**	2080 (100.0%)

A significant association was observed between occlusal status and dental appearance–related bullying (χ ^2^ = 694.38, df = 3, p < 0.001), with Cramer’s V = 0.578 (p < 0.001).

Children with anterior crossbite, particularly those with concomitant malocclusion, exhibited the highest prevalence of bullying compared to those with malocclusion alone or normal occlusion ( Table 3).

**Table 3.** Relationship between occlusal status and dental appearance-related bullying.

		Dental appearance–related bullying		
		Non-victims N (%)	Victims N (%)	Total N (%)
**Occlusal status**	**Normal occlusion**	702 (99.9%)	1 (0.1%)	703 (100.0%)
	**Malocclusion and anterior crossbite**	4 (12.9%)	27 (87.1%)	31 (100.0%)
	**Other malocclusion only**	562 (58.0%)	407 (42.0%)	969 (100.0%)
	**Anterior crossbite only**	97 (25.7%)	280 (74.3%)	377 (100.0%)
	**Total**	1365 (65.6%)	715 (34.4%)	2080 (100.0%)
**Chi-Square tests**	**Pearson Chi-Square **	***p*- Value**	694.380	
		**Degrees of Freedom(Df)**	3	
		**Asymptotic significance (2-sided)**	< 0.001 *	
**Symmetric measures**	**Phi**	***p*- Value**	0.578	
		**Approximate significance**	< 0.001 *	
	**Cramer’s V**	***p*- Value**	0.578	
		**Approximate significance**	< 0.001 *	

* Significant coefficient; no expected count less than 5.

Binary logistic regression, adjusted for age and gender, confirmed these findings. Older age was significantly associated with increased bullying victimization (Exp(B) = 1.556; 95% CI: 1.240–1.953; p < 0.001), while gender was not significant (Exp(B) = 1.007; 95% CI: 0.805–1.261; p = 0.949).

Compared with children with normal occlusion, those with both malocclusion and anterior crossbite had substantially increased bullying exposure, (Exp(B) = 0.000; 95% CI: 0.000–0.003; p < 0.001).

Children with anterior crossbite alone also showed significantly higher victimization risk (Exp(B) = 0.231; 95% CI: 0.176–0.303; p < 0.001), whereas those with malocclusion only did not differ significantly from the reference group (Exp(B) = 2.162; 95% CI: 0.735–6.360; p = 0.161). Findings are presented in Table 4.

**Table 4.** Logistic regression assessing the relationship between occlusal status and dental bullying.

Variables in the Equation		B	S.E.	Wald	df	Sig.	Exp (B)	95% C.I. for EXP(B)	
								Lower	Upper
**Age**		0.442	0.116	14.591	1	< 0.001*	1.556	1.240	1.953
**Gender**		0.007	0.115	0.004	1	0.949	1.007	0.805	1.261
**Occlusal status**	**Normal occlusion (reference)**			168.270	3	< 0.001*			
	**Malocclusion and anterior crossbite**	−7.686-	1.008	58.123	1	< 0.001*	0.000	0.000	0.003
	**Other malocclusion only**	0.771	0.550	1.963	1	0.161	2.162	0.735	6.360
	**Anterior crossbite only**	−1.465-	0.138	113.134	1	< 0.001*	0.231	0.176	0.303
**Constant**		−0.439-	0.442	0.989	1	0.320	0.644		

^1^ *A significant coefficient.

^2^ B: regression coefficient; SE: standard error; Wald: Wald chi-square; df: degrees of freedom; Sig: significance; Exp(B): odds ratio; 95% CI: 95% confidence interval

These results indicate that anterior crossbite, whether alone or combined with malocclusion, significantly increases bullying risk, whereas malocclusion without crossbite does not significantly influence victimization.

Among children with anterior crossbite, ordinal logistic regression revealed a clear dose–response relationship between ROMA severity and bullying severity.

Children requiring immediate treatment exhibited the highest bullying scores (Estimate = −3.761, 95% Confidence Interval: −4.730 to −2.793; p < 0.001), followed by those requiring observation (Estimate = −3.364, 95% Confidence Interval: −4.954 to −1.774; p < 0.001).

Children under routine follow-up showed no significant difference from the reference category (Estimate = −0.621, 95% Confidence Interval: −2.024 to 0.782; p = 0.385). Age (Estimate = 0.076; p = 0.649) and gender (Estimate = −0.477; p = 0.069) were not significantly associated with bullying severity. These findings indicate that bullying likelihood and severity are positively correlated with anterior crossbite severity and are independent of the child’s age and gender (see Table 5).

**Table 5.** Ordinal logistic regression analysis examining the dose–response relationship between the severity of anterior crossbite, as classified by the ROMA index, and the intensity of bullying exposure.

Threshold location		Estimate	Std. error	Wald	Df	Sig.	95% confidence interval	
							Lower bound	Upper bound
**Bullying severity**	**[score = 4.00]**	−10.624-	1.295	67.335	1	< 0.001 *	−13.161-	−8.086-
	**[score = 5.00]**	−8.415-	0.886	90.114	1	< 0.001 *	−10.153-	−6.678-
	**[score = 6.00]**	−1.993-	0.795	6.289	1	0.012 *	−3.550-	−0.435-
	**[score = 7.00]**	3.648	1.216	8.997	1	0.003 *	1.264	6.031
**Age**		0.076	0.166	0.207	1	0.649	−0.250-	0.402
**Gender**		−0.477-	0.263	3.298	1	0.069	−0.991-	0.038
**ROMA severity grade**	**4n**	−3.761-	0.494	57.958	1	< 0.001 *	−4.730-	−2.793-
	**3n**	−3.364-	0.811	17.202	1	< 0.001 *	−4.954-	−1.774-
	**2n**	−0.621-	0.716	0.753	1	0.385	−2.024-	0.782

SE: standard error; Wald: Wald chi-square; df: degrees of freedom; Sig: significance; CI: 95% confidence interval.

* A significant coefficient.

Teasing and mocking were the most common forms, reported by 97.2% of children with anterior crossbite only, 94.4% of those with both malocclusion and anterior crossbite, and 71.4% of those with malocclusion only. These differences were statistically significant (χ ^2^ = 50.79, df = 2, p < 0.001) with a moderate strength of association (Cramer’s V = 0.291).

Unpleasant nickname- calling showed a strong association with occlusal status (χ ^2^ = 96.38, df = 2, p < 0.001; Cramer’s V = 0.401), with prevalence varying significantly across groups. Social exclusion was less frequently reported, remained significantly correlated with occlusal status (χ ^2^ = 969.95, df = 2, p < 0.001; Cramer’s V = 0.342). Physical bullying occurred infrequently across all groups but still showed a statistically significant difference (χ ^2^ = 10.09, df = 2, p = 0.006; Cramer’s V = 0.130).

Bullying locations varied significantly among occlusal groups (χ ^2^ = 13.23, df = 6, p = 0.004; Cramer’s V = 0.133).

Children with both malocclusion and anterior crossbite most frequently experienced bullying on the playground (63.0%), followed by the classroom (25.9%) and the route to school (11.1%), with no reports from.

Those with malocclusion reported incidents only at the playground (42.9%), classroom (31.4%), route to school (14.3%), and multiple locations (11.4%). Children with anterior crossbite only experienced bullying predominantly on the playground (64.0%), in the classroom (14.6%), on the route to school (16.2%), and in multiple locations (5.1%).

Children with normal occlusion were excluded from the analysis, as dental appearance-related bullying was negligible in this group. Consequently, all descriptive and comparative analyses of bullying patterns focused exclusively on children with malocclusion and/or anterior crossbite ( Table 6).

**Table 6.** Patterns and locations of dental appearance-related bullying among children with varying occlusal characteristics.

Occlusal status			Bullying Patterns				Bullying location			
			Teasing and mocking N (%)	Unpleasant nicknames N (%)	Social exclusion N (%)	Physical bullying N (%)	ClassroomN(%)	Playground N (%)	Route to school N (%)	Multiple locations N (%)
	**Malocclusion and anterior crossbite**		51 (94.4%)	51 (94.4%)	9(16.7%)	1 (1.9%)	7 (25.9%)	17 (63.0%)	3(11.1%)	0(0.0%)
	**Other Malocclusion only**		25 (71.4%)	13 (37.1%)	2 (5.7%)	0 (0.0%)	11 (31.4%)	15 (42.9%)	5(14.3%)	4(11.4%)
	**Anterior crossbite only**		495(97.2%)	465(91.4%)	1 (0.2%)	0 (0.0%)	46 (14.6%)	201(64.0%)	51(16.2%)	16(5.1%)
	**Total**		571 (95.5%)	529 (88.5%)	1 (2.0%)	1 (0.2%)	64 (17.0%)	233(62.0%)	59(15.7%)	20(5.3%)
**Chi-Square Tests**		***p*- Value**	50.794	96.376	69.947	10.091	13.230			
		**Degrees of Freedom(Df)**	2	2	2	2	6			
		**Asymptotic Significance (2-sided)**	< 0.001 *	< 0.001 *	< 0.001 *	0.006 *	0.040 *			
**Symmetric Measures**	**Phi**	***p*- Value**	0.291	0.401	0.342	0.130	0.188			
		**Approximate Significance**	< 0.001 *	< 0.001 *	< 0.001 *	0.006 *	0.040 *			
	**Cramer’s V**	***p*- Value**	0.291	0.401	0.342	0.130	0.188			
		**Approximate Significance**	< 0.001 *	< 0.001 *	< 0.001 *	0.006 *	0.040 *			

* Significant coefficient

## Discussion

The study demonstrated that anterior crossbite extends beyond functional and aesthetic concerns to significantly impact children’s well-being in school settings. The prevalence of 19.6% in this study substantially exceeds global estimates off approximately 11% for mixed dentition, which are approximately 11%, placing this population in a higher-risk category and underscoring the urgency for early intervention. ^1^

Regional prevalence varies considerably, ranging from (5.5%) in Iraq to 14% in Palestine, with Egypt and Jordan reporting 10.1% and (6.8%) respectively ^35, 36^ Jordan. ^37, 38^

Notably, most existing studies examined older children than the current sample (ages 8–12 years), a critical developmental period characterized by ongoing dental and craniofacial changes. ^39^

During this stage, mild cases may spontaneously resolve or benefit from early treatment, making this age group essential for accurately assessing the overall disease burden. ^40^

The current study fills an important gap by providing both prevalence and severity estimates using the standardized ROMA index data previously lacking in early mixed dentition populations. These findings are vital for designing school-based screening initiatives and facilitating timely orthodontic referrals.

Dental appearance-related bullying affected 34.4% of the sample, highlighting a significant public health concern. Chronic childhood bullying exposure is well-documented to increase the risk of anxiety, depression, low self-esteem, and academic underperformance. ^41^

Longitudinal evidence demonstrates that bullying victims face substantially elevated odds of developing psychological disorders extending into adolescence and adulthood (Odds Ratio = 1.60; 95% Confidence Interval: 1.42–1.81). ^42^

By linking a clinically diagnosable occlusal condition to a prevalent psychosocial stressor, this study reframes anterior crossbite as more than a localized dental issue. It is a broader health determinant affecting mental well-being and academic success.

Statistical analyses revealed a robust association between malocclusion and bullying, Cramer’s V = 0.578, representing a substantial effect size with both clinical and epidemiological relevance. This finding aligns with prior research indicating that children with prominent anterior dental characteristics experience higher bullying rates than their peers without such features. ^43^ These results underscore the essential role of anterior facial appearance in social perception during a formative development period for identity formation and self-esteem, ^44^ supporting the integration of malocclusion as a psychosocial risk marker in school health programs.

The graded association between anterior crossbite severity and bullying corresponds with Bradford Hill’s criteria for establishing causality, particularly, the epidemiological dose–response principle. ^45^

Ordinal logistic regression analysis indicated that higher severity scores, particularly those necessitating immediate intervention, experienced significantly greater bullying exposure (p < 0.001). This dose- response pattern reinforces the causal inference and strengthens the importance of integrating severity assessments into school-based screening and early prevention strategies. Such targeted interventions could reduce both the clinical burden.

Age emerged as a significant predictor of bullying risk, while gender did not demonstrate a significant effect. This finding is consistent with existing studies indicating showing that bullying exposure typically increases during late primary and early adolescence. ^46^

While gender differences in bullying do occur, they generally reflect variations in bullying type rather than severity. ^47^ The age-related increase likely reflects a heightened sensitivity to social evaluation and physical appearance during this development stage, emphasizing the critical need for preventive interventions targeting older primary school children.

Verbal bullying, including teasing, derogatory comments, and name-calling, was substantially more prevalent than physical aggression, consistent with evidence that verbal and social forms of bullying dominate among children within this age group. ^48^

Although physical bullying occurred less frequently, the data suggest that appearance-based verbal harassment can escalate into more severe forms of aggression without early intervention. This finding underscores the importance of comprehensive, multifaceted prevention strategies within schools. Schools emerged as the primary bullying setting, underscoring the critical role of both environmental and organizational factors on aggressive behaviors. Meta-analytic evidence demonstrates that increased school supervision and anti-bullying initiatives significantly reduce bullying rates. ^49^

These findings support an integrated approach combining early orthodontic screening with school-based bullying prevention initiatives- a dual approach with substantial potential to improve both oral and psychosocial health outcomes.

These findings should be interpreted within the context of the study’s cross-sectional design, which limits causal inference. Additionally, unmeasured variables such as self-esteem and familial support factors may mediate the observed associations. Therefore, longitudinal, multicenter studies are needed to assess the long-term psychosocial benefits of early orthodontic intervention and to evaluate the cost-effectiveness of incorporating malocclusion screening into routine school health programs.

Anterior crossbite severity represents a clinically significant indicator with considerable social consequences. Future interventions should operate within a comprehensive public health framework that integrates oral health, psychological well-being, and educational environments.

A multi-level intervention combining early orthodontic care, school-based anti-bullying programs, and community education could significantly reduce the psychosocial burden associated with malocclusion during childhood and promote holistic child health and development.

## Conclusion

An anterior crossbite extends beyond dental aesthetics and function. It represents a significant psychosocial stressor in school environments, with effects that intensify as severity increases. This study establishes a clear dose–response relationship between anterior crossbite severity and bullying victimization, demonstrating clinical and social relevance.

Early identification of anterior crossbite severity during mixed dentition enables timely recognition of children at increased risk for psychosocial harm. Integrating orthodontic severity assessments into school health screening programs coupled with comprehensive anti-bullying initiatives offers a dual -intervention approach to safeguard children’s mental health and academic success.

**Corresponding author**

Correspondence to Farah M. Babakurd

## Ethics declarations

### Ethics approval and consent to participate

Ethical approval for this study was obtained from the Research Ethics Committee of the Faculty of Dentistry at Damascus University, Syria (IRB No. UDDS 2614_28042025/SRC_2320). Additionally, written consent was granted by the Ministry of Education (No. 4/443, 02/07/2025). The research adhered to the ethical principles of the Declaration of Helsinki (2013). Before participation, written informed consent was obtained from the parents or legal guardians of all children. The study’s reporting follows the STROBE (Strengthening the Reporting of Observational Studies in Epidemiology) guidelines.

### Consent for publication

All the parents or legal guardians of all children provided written informed consent for publication of anonymized data and findings.

## Data Availability

Underlying and extended data supporting the results of this study are deposited in the Open Science Framework (OSF) repository and can be accessed via the following DOI: https://doi.org/10.17605/OSF.IO/HW8KM. ^50^ Note: If the hyperlink does not activate directly in your document, please copy and paste the DOI into your web browser to access the dataset. This project contains the following underlying data: De-identified Excel Dataset (Child IDs).xlsx – Contains coded participant data without personal identifiers. Dataset Code Explanation.docx – Explains the coding system used in the Excel dataset. Parental Informed Consent Form.docx – Contains the consent form provided to participants. Participant Information Sheet.docx – Contains participant information details. Modified Dental Appearance-Related Bullying Questionnaire.docx – Contains the questionnaire used in the study. Data are available under the terms of the Creative Commons Zero “No rights reserved” data waiver (CC0 1.0 Universal). Note: The DOI link provides access to all underlying and extended files. No personal identifiers are included in any of the datasets to ensure participant confidentiality.
