# How Anterior Crossbite Severity Relates to Appearance-Based Bullying in School-Age Children: Evidence from the ROMA Index

**DOI:** 10.12688/f1000research.179073.2

**Published:** 2026-06-08

**Authors:** Farah M. Babakurd, Khaled Omar, Mayssoon Dashash

**Affiliations:** 1 Pediatric Dentistry Department, Faculty of Dentistry, Damascus University, Damascus, Syria; 2 Faculty of Informatics Engineering, Damascus University, Damascus, Syria; 3 Pediatric Dentistry Department, Faculty of Dentistry, Damascus University, Damascus, Syria

**Keywords:** Anterior crossbite, ROMA Index, dental appearance-related bullying, Olweus Bully/Victim Scale, Mixed dentition, Children.

## Abstract

**Introduction:**

This study examined the prevalence of anterior crossbite in school-age children, investigated the frequency of appearance- related bullying, and determined whether crossbite severity correlates with bullying exposure among children aged 8–12 years.

**Materials and Methods:**

A cross-sectional study involved 2,080 children from public schools in Damascus, using random cluster sampling.

Anterior crossbite and other occlusal issues were assessed using the ROMA Index. Dental appearance-related bullying was evaluated using a modified Olweus Bully/Victim Questionnaire; children reporting bullying two or more times monthly were classified as victims.

Bullying types—teasing, name-calling, social exclusion, and physical aggression—were documented along with occurrence settings. Severity scores were calculated by summing numerical codes for each bullying type. Relationships between occlusal characteristics and bullying were analyzed using Chi-square tests, Cramer’s V, and logistic regression, adjusting for age and gender.

**Results:**

Of 2,080 children aged 8 to 12 years, 19.6% had anterior crossbite, and 34.4%reported dental appearance-related bullying. Multivariable binary logistic regression revealed that all forms of malocclusion, particularly those involving an anterior crossbite, were associated with a significantly higher likelihood of experiencing bullying (p < 0.001), and that bullying intensity increased proportionally with crossbite severity. Teasing and mockery emerged as the most common forms of victimization, with rates and locations of such incidents—predominantly in playground settings—demonstrating significant variability across different occlusal characteristics, particularly when compared to the near-zero baseline observed in the normal occlusion group.

**Conclusion:**

Anterior crossbite severity serves as a meaningful correlate for appearance-based bullying in children, extending beyond simple dental concerns. Incorporating crossbite severity screening into school-based prevention programs offers a practical strategy to address both oral health and psychosocial well-being in childhood.

**Abbreviations**
ROMA index: Risk of Malocclusion Assessment Index
OHRQoL: oral health–related quality of life

## Introduction

Malocclusion is among the most prevalent oral health conditions globally, affecting approximately 56% of the population. Prevalence varies considerably across populations and diagnostic criteria. ^1^ Beyond functional consequences, malocclusion carries a significant psychosocial burden, particularly during childhood—a period of increased sensitivity to peer judgments and appearance-based evaluation. ^2– 4^

A meta-analysis of 40 cross-sectional studies demonstrated significantly reduced oral health–related quality of life (OHRQoL) in children with malocclusion. Children with malocclusion were 1.74 times more likely to experience OHRQoL impairment than unaffected peers. ^5^

Severity showed a dose-response relationship: Children with normal or mild malocclusion were 56% less likely to experience quality-of-life impairments than those with severe malocclusion. ^6^

Despite robust evidence linking malocclusion to compromised quality of life, the association between malocclusion and school bullying remains contested. A recent systematic review found that 88% of studies reported a positive correlation between dentofacial anomalies and victimization. ^7^

However, a subsequent meta-analysis yielded inconclusive results, reflecting heterogeneity across studies. ^8^

Bullying, defined as recurrent aggressive behavior within a power- imbalanced relationship, carries serious psychological and academic consequences, including reduced self-esteem, academic decline, and heightened risk of anxiety and depression. ^9^

Appearance- related bullying specifically affects 7% to 47.8% of children globally, ^10, 11^ underscoring the social significance of visible dentofacial characteristics in school settings. ^12^

The mixed dentition stage (8–12 years) represents a critical window during which peer awareness intensifies, magnifying psychosocial impacts of visible dentofacial anomalies such as anterior crossbite. ^3^

This type of sagittal malocclusion, specifically anterior crossbite, occurs in approximately 11% of the population. ^1^ If left untreated, an anterior crossbite can compromise the perception of the facial profile, cause functional mandibular displacement, lead to abnormal incisal wear, and worsen skeletal Class III patterns. ^13^

Beyond functional consequences, anterior crossbite affects dental aesthetics during speech and smiling; its psychosocial effects correlate with severity. ^14^ However, limited research examines the relationship between anterior crossbite severity and appearance-related bullying, especially in Middle Eastern populations. Understanding these psychosocial dimensions is important for timely orthodontic referral and early intervention.

The Risk of Malocclusion Assessment (ROMA) Index provides an objective and standardized, reproducible classification of occlusal disorders, including anterior crossbite. ^15^ The modified Olweus Bully/Victim Questionnaire enables systematic evaluation of bullying exposure, appearance- related victimization, severity, patterns, and contexts. ^16, 17^

Combining objective clinical severity assessment with validated psychosocial measurement minimizes subjective bias and strengthens methodological rigor.

This study addressed a significant knowledge gap: the lack of severity-based analyses of anterior crossbite and bullying, and the absence of data from Syrian populations. The study aimed to ascertain the prevalence of anterior crossbite in children aged 8–12 years using the ROMA Index, estimate the prevalence of appearance-related bullying using the modified Olweus questionnaire, analyze the association between anterior crossbite severity and bullying exposure while controlling for age and gender, and describe bullying patterns and contexts.

The findings are expected to deepen the understanding of the psychosocial dimensions of anterior crossbite during mixed dentition, inform early orthodontic intervention decisions integrating functional and psychological considerations, and support integration of orthodontic care into school-based bullying prevention programs.

## Materials and methods

This cross-sectional analytical study received ethical approval from the Research Ethics Committee at the Faculty of Dentistry, University of Damascus (IRB No. UDDS 2614_28042025/SRC_2320) and authorization from the Ministry of Education (No. 4/443, 02/07/2025).

The study followed the Declaration of Helsinki (2013), ^18^ and written informed consent was obtained from parents or legal guardians of all participating children.

Data collection occurred between July and December 2025, and the research complies with the STROBE guidelines for observational studies. ^19^

### Sampling and study population

A cluster random sampling design was employed across public schools in Damascus, with schools designated as clusters. Children were assessed in private rooms within each school under the teacher’s supervision to ensure privacy and accuracy. ^20^

According to the Directorate of Education, the first cycle included 56,772 boys and 53,761 girls, totaling 110,533 children. Sample size calculation for determining anterior crossbite prevalence (±5% precision, and of 95% confidence level) used the reported global prevalence of anterior crossbite (11%) ^1^ and the standard formula for cross-sectional studies ^21^:

$$n=(Z^{2*}\;P^{*}\;(1-P))/D^{2}$$

With a 95% confidence level ( *Z*) and a 2% margin of error ( *D*), this yielded an initial estimate of approximately 150 children.

To assess the association between anterior crossbite and dental appearance-related bullying, global prevalence rates range from 7% to 47.8%. ^10, 11^ However, no published data exist for children with anterior crossbite in Asia or Syria. Conservative estimates were therefore adopted (P1 ≈ 30% for affected children, P2 ≈ 15% for unaffected) for binary outcome analysis using Chi-square or Odds Ratio methods, maintaining 80% power and 95% confidence level. ^22^ This calculation provided an initial estimate of approximately 236 children. Accounting for a potential 10–15% non-response rate of, ^23^ the sample size was adjusted to approximately 270 children.

Given that the study aimed to estimate the prevalence of anterior crossbite, and the prevalence of bullying, analyze associations within subgroups (such as age, gender, and crossbite severity), and perform logistic regression analyses, the final sample was expanded to 2,080 children to ensure adequate statistical power. ^24^

In light of the multistage cluster sampling design employed, with schools designated as clusters, it is essential to account for the Design Effect (Deff) to address variance inflation and the inherent homogeneity within the clusters. ^25^ A conservative design effect of 2.5 was assumed, based on epidemiological benchmarks for school surveys, ^26^ resulting in an adjusted sample size of approximately 675 children (270 multiplied by 2.5) required to preserve statistical power for simple associations.

Moreover, given that the primary aim of this study extends beyond bivariate associations to encompass complex multivariable modeling—specifically, ordinal logistic regression analyses—there is a need to examine the dose-response relationship among ROMA severity subgroups (as presented in Table 5) as well as to conduct sub-analyses that control for age and gender (as demonstrated in Table 4). Consequently, a considerably larger sample size was necessitated to prevent model overfitting, ensure adequate cell frequencies across all cross-tabulations, and mitigate high standard errors. ^27– 29^ To attain robust statistical power (1-β = 0.90) capable of detecting small expected effect sizes (f ^2^ = 0.02) within these multi-categorical subgroups, the final study population was expanded to 2,080 children. ^30^ This augmentation is critical to ensuring maximum epidemiological precision, achieving narrow confidence intervals, and maintaining the stability of the multivariable regression coefficients.

### Clinical assessment of malocclusion and other occlusal features

A single certified examiner (FB) supported by two school health department staff members performed all clinical examinations. To evaluate intra-examiner reliability, 10% of participants underwent repeat examinations, spaced by 20 to 30-minutes. ^31^ Cohen’s kappa values ranged from 0.85 to 0.95, indicating excellent agreement. ^32^

Occlusion characteristics were classified using the ROMA Index, a standardized grading system that categorizes malocclusions by severity and orthodontic treatment need. The index defines three grades ^15^:

Grade 2 N: Mild malocclusion requiring routine monitoring.

Grade 3 N: Moderate malocclusion with clear need for orthodontic treatment.

Grade 4 N: Severe malocclusion requiring urgent or immediate orthodontic intervention.

Four key occlusal traits were evaluated and graded according to clinical severity and treatment urgency:

• Anterior crossbite: one or more maxillary teeth occluding lingually to the corresponding mandibular teeth in centric occlusion.
• Anterior open bite: Absence of vertical overlap between opposing anterior teeth.
• Deep bite: Excessive vertical overlap of anterior teeth.
• Increased overjet: Horizontal protrusion of maxillary incisors.

All examinations followed standardized clinical protocols. When precise measurements were required to support. ^15^

A calibrated Williams periodontal probe (1–10 mm increments) was used to ensure consistent and reproducible measurements. ^33^

### Assessment of dental appearance-related bullying

Researchers adapted the Olweus Bully/Victim Questionnaire to measure bullying related to dental appearance. ^16, 17^

The modified instrument evaluated the frequency and types of bullying incidents over a two-month reference period. Children were classified as victims if they experienced two or more bullying incidents per month, consistent with prior research. ^34^

The questionnaire assessed multiple forms of dental appearance-related bullying: teasing, name-calling, social exclusion, and physical aggression. Each response was coded numerically (Yes = 1, No = 2) to generate a bullying severity score ranging from 4 to 8 for each participant, with lower scores indicating greater bullying exposure. Researchers also recorded where bullying occurred (classroom, schoolyard, street, or multiple locations).

Before the main study with 2,080 children, a pilot study with 100 randomly selected participants tested the validity and reliability of the questionnaire, approximately 5% of the main sample. The pilot phase evaluated item clarity, validity, and reliability of the modified instrument. ^35^

Results demonstrated robust psychometric properties. Face validity was confirmed by 90% of expert reviewers. The Content Validity Index (CVI) was 0.92, ^36^ internal consistency (Cronbach’s alpha) was 0.85, ^37^ and test-retest reliability measured at two weeks yielded an Intraclass Correlation Coefficient (ICC) of 0.88. ^38, 39^ These findings confirmed the questionnaire’s reliability and stability of use in the main study. Refinements were made to items as needed ensuring the modified instrument ^40^ is aligned with international research standards.

### Inclusion and exclusion criteria

Children aged 8 to 12 years enrolled in public schools were eligible for participation if they could complete both the questionnaire and clinical examination and had obtained parental consent.

The study excluded individuals with chronic medical conditions or disabilities, prior orthodontic treatment, prolonged school absences, or learning difficulties that could affect their ability to respond reliably to the questionnaire.

### Statistical analysis

Data analysis was performed using SPSS software. Descriptive statistics summarized demographic characteristics, the prevalence of anterior crossbite, and the frequency of dental appearance-related bullying. Categorical variables were reported as frequencies and percentages, while continuous variables were presented as means and standard deviations.

Chi-square tests were used to examine associations between occlusal status and bullying, with Cramer’s V utilized to measure the strength of these associations. Post hoc pairwise comparisons identified specific differences between groups. Ordinal logistic regression assessed how anterior crossbite severity influenced bullying severity, while binary logistic regression determined the odds of bullying victimization across different occlusal groups after adjusting for age and gender. The locations and patterns of bullying incidents were analyzed descriptively using Chi-square and Cramer’s V comparisons. All statistical tests were two-tailed with a significance level of p < 0.05.

## Results

A total of 2,080 children aged 8–12 years were included.

Among the participants, 408 children (19.6%) presented with an anterior crossbite. Of these cases, 15% required immediate intervention, 2.6% showed signs of malocclusion that could persist or worsen, and2% required routine occlusal monitoring. Findings are presented in Table 1.

**Table 1.** Prevalence of anterior crossbite among children aged 8–12 years, categorized by severity and required intervention according to the ROMA Index.

Anterior Crossbite	N(%)	ROMA Grade	N(%)
**Present**	408 (19.6%)	**4n: Crossbite > 2 mm**	312 (15.0%)
		**3n: Crossbite > 1 mm**	55 (2.6%)
		**2n: Crossbite < 1 mm**	41 (2.0%)
**Absent**	167 (80.4%)		
**Total**	208 (100.0%)		

The modified Olweus Bully/Victim Questionnaire identified 34.4% of the children as victims of dental appearance- related bullying. Victimization was defined in accordance with established Olweus criteria, whereby children who reported experiencing bullying two or more times per month were classified as victims. In contrast, those who reported no instances of bullying or only occasional occurrences were categorized as non-victims (see Table 2).

**Table 2.** Prevalence of dental appearance-related school bullying among children aged 8 to 12 years with mixed dentition.

Modified Olweus Bully/Victim Questionnaire	N(%)
**Victims**	715 (34.4%)
**Non-victims **	1365 (65.6%)
**Total**	2080 (100.0%)

A significant association was observed between occlusal status and dental appearance–related bullying (χ ^2^ = 694.38, df = 3, p < 0.001), with Cramer’s V = 0.578 (p < 0.001).

Children with anterior crossbite, particularly those with concomitant malocclusion, exhibited the highest prevalence of bullying compared to those with malocclusion alone or normal occlusion ( Table 3).

**Table 3.** Relationship between occlusal status and dental appearance-related bullying.

		Dental appearance–related bullying		
		Non-victims N (%)	Victims N (%)	Total N (%)
**Occlusal status**	**Normal occlusion**	702 (99.9%)	1 (0.1%)	703 (100.0%)
	**Malocclusion and anterior crossbite**	4 (12.9%)	27 (87.1%)	31 (100.0%)
	**Other malocclusion only**	562 (58.0%)	407 (42.0%)	969 (100.0%)
	**Anterior crossbite only**	97 (25.7%)	280 (74.3%)	377 (100.0%)
	**Total**	1365 (65.6%)	715 (34.4%)	2080 (100.0%)
**Chi-Square tests**	**Pearson Chi-Square **	***p*- Value**	694.380	
		**Degrees of Freedom(Df)**	3	
		**Asymptotic significance (2-sided)**	< 0.001 *	
**Symmetric measures**	**Phi**	***p*- Value**	0.578	
		**Approximate significance**	< 0.001 *	
	**Cramer’s V**	***p*- Value**	0.578	
		**Approximate significance**	< 0.001 *	

* Significant coefficient; no expected count less than 5.

Binary logistic regression, adjusted for age and gender, corroborated these findings (refer to Table 4). The analysis revealed that older age is significantly associated with an increased likelihood of experiencing bullying victimization (EXP(B) = 1.239, 95% C.I.: 1.111-1.383, P < 0.001). Conversely, gender did not significantly affect the outcome (EXP(B) = 0.822, 95% C.I.: 0.642-1.054, P = 0.123).

**Table 4.** Logistic regression assessing the relationship between occlusal status and dental bullying.

Variables in the Equation	B	S.E.	Wald	df	Sig.	Exp (B)	95% C.I. for EXP(B)	
							Lower	Upper
**Age (Continuous)**	0.215	0.056	14.697	1	<0.001 *	1.239	1.111	1.383
**Gender (Ref: Females)**	-0.196	0.127	2.385	1	0.123	0.822	0.642	1.054
**Occlusal status**			168.617	3	<0.001 *			
**Normal occlusion (reference)**					1.000			
**Malocclusion and anterior crossbite**	8.451	1.136	55.355	1	<0.001 *	4678.491	504.983	43344.549
**Other Malocclusion only**	6.210	1.003	38.340	1	<0.001 *	497.615	69.700	3552.681
**Anterior crossbite only**	7.678	1.008	58.011	1	<0.001 *	2160.414	299.538	15581.948
**Constant**	-8.503	1.127	56.954	1	<0.001 *	0.001		

* A significant coefficient.

B: regression coefficient; SE: standard error; Wald: Wald chi-square; df: degrees of freedom; Sig: significance; Exp(B): odds ratio; 95% CI: 95% confidence interval.

In comparison to children exhibiting normal occlusion, those with both malocclusion and anterior crossbite demonstrated an extraordinarily elevated likelihood of being bullied (EXP(B) = 4678.491, 95% C.I.: 504.983-43344.549, P < 0.001). Likewise, children presenting with anterior crossbite alone encountered an immensely heightened risk of victimization (EXP(B) = 2160.414, 95% C.I.: 299.538-15581.948, P < 0.001). It is important to note that children with other types of malocclusion also revealed a significantly greater risk of bullying when compared to the reference group (EXP(B) = 497.615, 95% C.I.: 69.700-3552.681, P < 0.001).

These findings underscore that all forms of malocclusion, particularly those associated with anterior crossbite, substantially elevate the risk of appearance-based bullying among school-age children.

Among children with anterior crossbite, ordinal logistic regression revealed a clear dose–response relationship between ROMA severity and bullying severity.

Children requiring immediate treatment exhibited the highest bullying scores (Estimate = −3.761, 95% Confidence Interval: −4.730 to −2.793; p < 0.001), followed by those requiring observation (Estimate = −3.364, 95% Confidence Interval: −4.954 to −1.774; p < 0.001).

Children under routine follow-up showed no significant difference from the reference category (Estimate = −0.621, 95% Confidence Interval: −2.024 to 0.782; p = 0.385). Age (Estimate = 0.076; p = 0.649) and gender (Estimate = −0.477; p = 0.069) were not significantly associated with bullying severity.

To facilitate accurate interpretation for readers unfamiliar with the modified Olweus scoring method, it is important to note that lower scores on this scale denote more severe bullying exposure. Therefore, the negative ordinal logistic regression coefficients confirm that as anterior crossbite clinical severity increases (higher ROMA grades), children experience a statistically significant and proportional progression toward greater bullying exposure (p < 0.001). Consequently, these findings indicate that bullying likelihood and severity are positively correlated with anterior crossbite severity and are independent of the child’s age and gender (see Table 5).

**Table 5.** Ordinal logistic regression analysis examining the dose–response relationship between the severity of anterior crossbite, as classified by the ROMA index, and the intensity of bullying exposure.

Threshold location		Estimate	Std. error	Wald	Df	Sig.	95% confidence interval	
							Lower bound	Upper bound
**Bullying severity**	**[score = 4.00]**	−10.624-	1.295	67.335	1	< 0.001 *	−13.161-	−8.086-
	**[score = 5.00]**	−8.415-	0.886	90.114	1	< 0.001 *	−10.153-	−6.678-
	**[score = 6.00]**	−1.993-	0.795	6.289	1	0.012 *	−3.550-	−0.435-
	**[score = 7.00]**	3.648	1.216	8.997	1	0.003 *	1.264	6.031
**Age**		0.076	0.166	0.207	1	0.649	−0.250-	0.402
**Gender**		−0.477-	0.263	3.298	1	0.069	−0.991-	0.038
**ROMA severity grade**	**4n**	−3.761-	0.494	57.958	1	< 0.001 *	−4.730-	−2.793-
	**3n**	−3.364-	0.811	17.202	1	< 0.001 *	−4.954-	−1.774-
	**2n**	−0.621-	0.716	0.753	1	0.385	−2.024-	0.782

SE: standard error; Wald: Wald chi-square; df: degrees of freedom; Sig: significance; CI: 95% confidence interval.

* A significant coefficient.

*Note:* It is important to note that within the revised Olweus scoring methodology, lower numerical scores paradoxically indicate a higher degree of exposure and greater severity of bullying victimization. Thus, the direction of the regression coefficients must be interpreted in light of this inverse scaling, whereby an elevated ROMA grade corresponds to diminished clinical scores, reflecting more intense experiences of bullying.

Regarding the specific patterns of victimization, teasing and mocking emerged as the predominant forms of bullying across the study sample, reported by 72.9% of children with anterior crossbite only, 87.1% of those with both malocclusion and anterior crossbite, and 2.6% of those with other malocclusion only. In stark contrast, only one child (0.1%) in the normal occlusion group reported experiences of teasing. These differences among the four groups were statistically significant (χ ^2^ = 1331.967, df = 3, p < 0.001), exhibiting a robust association (Cramer’s V = 0.792).

Furthermore, the incidence of derogatory nickname-calling demonstrated a strong correlation with occlusal status (χ ^2^ = 1275.224, df = 3, p < 0.001; Cramer’s V = 0.775). This form of bullying was prevalent among children with both malocclusion and anterior crossbite (87.1%) and those with anterior crossbite only (66.6%), while it was absent (0.0%) in the normal occlusion group. Although social exclusion was reported less frequently overall, it still exhibited a significant correlation with occlusal status (χ ^2^ = 453.814, df = 3, p < 0.001; Cramer’s V = 0.462). Physical bullying occurred infrequently across all groups but revealed a statistically significant difference (χ ^2^ = 67.483, df = 3, p < 0.001; Cramer’s V = 0.178), with incidents exclusively occurring in the combined malocclusion and anterior crossbite group (3.2%).

The locations of bullying incidents also displayed significant variability among the four occlusal groups (χ ^2^ = 17.354, df = 9, p = 0.043; Cramer’s V = 0.129). Children with normal occlusion functioned as a baseline comparative anchor, with only one child indicating that an incident occurred strictly within the classroom (100.0%). For the remaining clinical groups, the playground consistently represented the primary location for bullying. Specifically, children with both malocclusion and anterior crossbite frequently experienced bullying on the playground (63.0%), followed by the classroom (25.9%) and the route to school (11.1%). Those with other malocclusions primarily reported incidents occurring on the playground (44.0%), followed by the classroom (32.0%), the route to school (16.0%), and multiple locations (8.0%). Similarly, children with anterior crossbite only predominantly experienced bullying on the playground (62.4%), followed by the route to school (17.4%), the classroom (14.6%), and multiple locations (5.6%) ( Table 6).

**Table 6.** Patterns and locations of dental appearance-related bullying among children with varying occlusal characteristics.

			Bullying patterns				Bullying location			
			Teasing and mocking N (%)	Unpleasant nicknames N (%)	Social exclusion N (%)	Physical bullying N (%)	Classroom N (%)	Playground N (%)	Route to school N (%)	Multiple locations N (%)
**Occlusal status**	Normal occlusion *n* = 703		1 (0.1%)	0 (0.0%)	0 (0.0%)	0 (0.0%)	1 (100.0%)	0 (0.0%)	0 (0.0%)	0 (0.0%)
	Malocclusion and anterior crossbite n = 31		27 (87.1%)	27 (87.1%)	9 (29.0%)	1 (3.2%)	7 (25.9%)	17 (63.0%)	3 (11.1%)	0 (0.0%)
	Other Malocclusion only *n* = 969		25 (2.6%)	13 (1.3%)	2 (0.2%)	0 (0.0%)	8 (32.0%)	11 (44.0%)	4 (16.0%)	2 (8.0%)
	Anterior crossbite only *n* = 377		275 (72.9%)	251 (66.6%)	1 (0.3%)	0 (0.0%)	42 (14.6%)	179 (62.4%)	50 (17.4%)	16 (5.6%)
	Total *n* = 2080		328 (15.8%)	291 (14.0%)	12 (0.6%)	1 (0.05%)	58 (17.1%)	207 (60.9%)	57 (16.9%)	18 (5.3%)
**Chi-Square Tests**		***p*- Value**	1331.967	1275.224	453.814	67.483	17.354			
		**Degrees of Freedom (Df)**	3	3	3	3	9			
		**Asymptotic Significance (2-sided)**	<0.001 *	<0.001 *	<0.001 *	<0.001 *	0.043 *			
**Symmetric Measures**	**Phi**	***p*- Value**	0.792	0.775	0.462	0.178	0.223			
		**Approximate Significance**	<0.001 *	<0.001 *	<0.001 *	<0.001 *	0.043 *			
	** Cramer’s V**	***p*- Value**	0.792	0.775	0.462	0.178	0.129			
		**Approximate Significance**	<0.001 *	<0.001 *	<0.001 *	<0.001 *	0.043 *			

* Significant coefficient.

## Discussion

The study revealed that malocclusion, particularly anterior crossbite, has implications that extend beyond functional and aesthetic considerations, significantly affecting the psychosocial well-being of children within educational environments. The prevalence of 19.6% in this study substantially exceeds global estimates of approximately 11% for mixed dentition. This finding underscores a heightened epidemiological burden in this specific Syrian school-age population and emphasizes the critical importance of early intervention in clinical practice. ^1^

Regional prevalence varies considerably, ranging from (5.5%) in Iraq to 14% in Palestine, with Egypt and Jordan reporting 10.1% and (6.8%) respectively ^41, 42^ Jordan. ^43, 44^

Notably, most existing studies examined older children than the current sample (ages 8–12 years), a critical developmental period characterized by ongoing dental and craniofacial changes. ^45^

During this stage, mild cases may spontaneously resolve or benefit from early treatment, making this age group essential for accurately assessing the overall occlusal burden. ^46^

The current study fills an important gap by providing both prevalence and severity estimates using the standardized ROMA index data previously lacking in early mixed dentition populations. These findings are vital for designing school-based screening initiatives and facilitating timely orthodontic referrals.

Dental appearance-related bullying affected 34.4% of the sample, highlighting a significant public health concern. Chronic childhood bullying exposure is well-documented to increase the risk of anxiety, depression, low self-esteem, and academic underperformance. ^47^

Longitudinal evidence demonstrates that bullying victims face substantially elevated odds of developing psychological disorders extending into adolescence and adulthood (Odds Ratio = 1.60; 95% Confidence Interval: 1.42–1.81). ^48^

By linking a clinically diagnosable occlusal condition to a prevalent psychosocial stressor, this study reframes anterior crossbite as more than a localized dental issue. It is a broader health determinant affecting mental well-being and academic success.

Statistical analyses revealed a robust association between malocclusion and bullying, Cramer’s V = 0.578, representing a substantial effect size with both clinical and epidemiological relevance. This finding aligns with prior research indicating that children with prominent anterior dental characteristics experience higher bullying rates than their peers without such features. ^49^ These results underscore the essential role of anterior facial appearance in social perception during a formative development period for identity formation and self-esteem, ^50^ supporting the integration of malocclusion as a psychosocial correlate in school health programs.

The graded relationship between anterior crossbite severity and bullying demonstrates a clear epidemiological dose–response pattern. Ordinal logistic regression analysis indicated that higher severity scores, particularly those necessitating immediate intervention, were associated with significantly greater bullying exposure (p < 0.001). This dose-response pattern strongly reinforces the strength of the association and underscores the clinical importance of integrating severity assessments into school-based screening and early interceptive strategies, which could significantly reduce the psychosocial burden.

Age emerged as a significant predictor of bullying risk, with each one-year increase in age elevating the likelihood of victimization by 23.9% (EXP(B) = 1.239, p < 0.001), while gender did not demonstrate a significant effect. This finding is consistent with existing studies showing that bullying exposure typically increases during late primary and early adolescence. ^51^

While gender differences in bullying do occur, they generally reflect variations in bullying type rather than severity. ^52^ The age-related increase likely reflects a heightened sensitivity to social evaluation and physical appearance during this development stage, emphasizing the critical need for preventive interventions targeting older primary school children.

Verbal bullying, including teasing, derogatory comments, and name-calling, was substantially more prevalent than physical aggression, consistent with evidence that verbal and social forms of bullying dominate among children within this age group. ^53^

The incorporation of the normal occlusion group as a baseline control in this study substantially reinforces the significance of dental aesthetics in relation to victimization. While appearance-based bullying and negative social interactions were virtually non-existent among children with normal occlusion (0.1%), there was a marked increase in such behaviors among children exhibiting malocclusion and anterior crossbite. This finding indicates that the structural dental anomaly is the primary target of peer aggression.

Although physical bullying occurred less frequently, the data suggest that appearance-based verbal harassment can escalate into more severe forms of aggression without early intervention. This finding underscores the importance of comprehensive, multifaceted prevention strategies within schools. Schools, particularly school playgrounds, emerged as the primary bullying setting, underscoring the critical role of both environmental and organizational factors on aggressive behaviors. This is further evidenced by our baseline anchor case, where the only reported incident of bullying in the healthy group occurred strictly within the school environment. Meta-analytic evidence demonstrates that increased school supervision and anti-bullying initiatives significantly reduce bullying rates. ^54^

These findings support an integrated approach combining early orthodontic screening with school-based bullying prevention initiatives- a dual approach with substantial potential to improve both oral and psychosocial health outcomes.

These findings should be interpreted within the context of the study’s cross-sectional design, which prohibits any causal inferences regarding the relationship between anterior crossbite and bullying victimization. While robust statistical associations have been established, the correlations observed may be influenced by unmeasured confounding variables that were not included or adjusted for in our regression models. Potential confounders include baseline self-esteem, pre-existing psychological vulnerabilities, family support structures, and socioeconomic status, all of which may confound or mediate the observed relationship. Therefore, longitudinal, multicenter studies are needed to assess the long-term psychosocial benefits of early orthodontic intervention and to evaluate the cost-effectiveness of incorporating malocclusion screening into routine school health programs.

Anterior crossbite severity represents a clinically significant correlate with substantial social repercussions. Future interventions should operate within a comprehensive public health framework that integrates oral health, psychological well-being, and educational environments.

A multi-level intervention combining early orthodontic care, school-based anti-bullying programs, and community education could significantly reduce the psychosocial burden associated with malocclusion during childhood and promote holistic child health and development.

## Conclusion

An anterior crossbite extends beyond dental aesthetics and function. It represents a significant psychosocial stressor in school environments, with effects that intensify as severity increases. This study demonstrates a clear dose–response relationship between anterior crossbite severity and bullying victimization, demonstrating clinical and social relevance.

Early identification of anterior crossbite severity during mixed dentition enables timely recognition of children with an increased likelihood of experiencing psychosocial harm. Integrating orthodontic severity assessments into school health screening programs coupled with comprehensive anti-bullying initiatives offers a dual -intervention approach to safeguard children’s mental health and academic success.

**Corresponding author**

Correspondence to Farah M. Babakurd

## Ethics declarations

### Ethics approval and consent to participate

Ethical approval for this study was obtained from the Research Ethics Committee of the Faculty of Dentistry at Damascus University, Syria (IRB No. UDDS 2614_28042025/SRC_2320). Additionally, written consent was granted by the Ministry of Education (No. 4/443, 02/07/2025). The research adhered to the ethical principles of the Declaration of Helsinki (2013). Before participation, written informed consent was obtained from the parents or legal guardians of all children. The study’s reporting follows the STROBE (Strengthening the Reporting of Observational Studies in Epidemiology) guidelines.

### Consent for publication

All the parents or legal guardians of all children provided written informed consent for publication of anonymized data and findings.

## Data Availability

Underlying and extended data supporting the results of this study are deposited in the Open Science Framework (OSF) repository and can be accessed via the following DOI: https://doi.org/10.17605/OSF.IO/HW8KM. ^55^ Note: If the hyperlink does not activate directly in your document, please copy and paste the DOI into your web browser to access the dataset. This project contains the following underlying data: De-identified Excel Dataset (Child IDs).xlsx – Contains coded participant data without personal identifiers. Dataset Code Explanation.docx – Explains the coding system used in the Excel dataset. Parental Informed Consent Form.docx – Contains the consent form provided to participants. Participant Information Sheet.docx – Contains participant information details. Modified Dental Appearance-Related Bullying Questionnaire.docx – Contains the questionnaire used in the study. Data are available under the terms of the Creative Commons Zero “No rights reserved” data waiver (CC0 1.0 Universal). Note: The DOI link provides access to all underlying and extended files. No personal identifiers are included in any of the datasets to ensure participant confidentiality.
