# Supplementary material for: How Anterior Crossbite Severity Relates to Appearance-Based Bullying in School-Age Children: Evidence from the ROMA Index
Source: F1000Res. 2026 Jun 23;15:590. Originally published 2026 Apr 19. [Version 3] doi: 10.12688/f1000research.179073.3 (PMC13560802; doi:10.12688/f1000research.179073.3)
Supplement: Peer review history

# Reviewer report: Reviewer response for version 3

**DOI:** 10.5256/f1000research.204078.r494777

**Published:** 2026-08-19

**Authors:** Supriya Nambiar

**Affiliations:** 1 Manipal College of Dental Sciences Mangalore, Manipal Academy of Higher Education, Manipal, kart, India

Summary:

 The study investigates the association between anterior crossbite severity and appearance-related bullying in 2,080 Syrian schoolchildren aged 8–12, using a cross-sectional design with the ROMA Index and a modified Olweus Bully/Victim Questionnaire. It reports a dose-response relationship between crossbite severity and bullying intensity.

 Strengths:

 1. Large sample size:  (n=2,080) improves statistical power and generalizability within the sampled population.

 2. Random cluster sampling is appropriate for school-based population studies and helps reduce selection bias.

**3. **Use of validated instruments (ROMA Index, modified Olweus questionnaire) strengthens measurement validity.

 4. Appropriate statistical approach - combining Chi-square/Cramer's V with multivariable logistic regression (adjusted for age and gender) allows both bivariate screening and confounder-adjusted estimates.

 5. Clinically relevant conclusion — linking orthodontic screening to psychosocial prevention programs gives the findings translational value.

 Concerns: 

 1. Confounders: Adjustment is limited to age and gender. Socioeconomic status, other visible physical traits, self-esteem, and school-level factors are plausible confounders that could substantially affect the appearance-bullying link and are not addressed.

 5. Selection/reporting bias: Self-reported bullying via questionnaire in children can be subject to recall and social desirability bias; this isn't acknowledged as a limitation .

 Minor/Technical Issues:

 1. Formatting errors present in the text: a stray quotation mark ("t"hose) and missing space ("34.4%reported") — these read as transcription/typesetting errors and should be corrected before submission.

 2. "ROMA Index" should be spelled out at first use (Reduced Orthodontic Malocclusion Assessment or equivalent, depending on which index is meant) for readers unfamiliar with the acronym.

 3. Consider specifying whether the 34.4% bullying prevalence is among the full sample or specific to the crossbite subgroup — as written it's ambiguous.

 4. "Near-zero baseline" in the normal occlusion group is a strong claim — worth stating the actual percentage for transparency.

 Recommendation

**Minor Revision.** The study addresses a meaningful and underexplored intersection of pediatric dentistry and psychosocial health, but the paper would benefit from the mentioned suggestions .

Is the work clearly and accurately presented and does it cite the current literature?

Yes

If applicable, is the statistical analysis and its interpretation appropriate?

I cannot comment. A qualified statistician is required.

Are all the source data underlying the results available to ensure full reproducibility?

Yes

Is the study design appropriate and is the work technically sound?

Yes

Are the conclusions drawn adequately supported by the results?

Yes

Are sufficient details of methods and analysis provided to allow replication by others?

Yes

Reviewer Expertise:

Orthodontics

I confirm that I have read this submission and believe that I have an appropriate level of expertise to confirm that it is of an acceptable scientific standard, however I have significant reservations, as outlined above.

# Reviewer report: Reviewer response for version 3

**DOI:** 10.5256/f1000research.204078.r485887

**Published:** 2026-08-18

**Authors:** Ajay Kubavat, Ravi Anthony

**Affiliations:** 1 Orthodontics, Austin Institute of Dental Medicine, Austin, Texas, USA; 2 Sankalchand Patel University, Visnagar, Gujarat, India

SUMMARY OF THE ARTICLE

 This cross-sectional study of 2,080 children aged 8-12 years attending public schools in Damascus reports a 19.6% prevalence of anterior crossbite graded with the ROMA Index, a 34.4% prevalence of dental appearance-related bullying measured with a modified Olweus Bully/Victim Questionnaire, and a graded association between crossbite severity and bullying exposure. The research question is worthwhile and under-studied in this region, the sample is large, both the clinical index and the psychosocial instrument are standardised, intra-examiner reliability is documented, the underlying data are openly deposited on OSF, and the authors have responded constructively to the earlier review rounds - the expanded Limitations section in version 3 is a real improvement.

 My reservations concern the internal consistency of the reported counts, the handling of separation in the logistic models, and the fact that the cluster design is used to justify the sample size but is not carried through to the analysis. All of these are addressable, and I would be glad to look at a revised version.

 MAJOR POINTS

 1. Tables 3 and 6 cannot both be correct.

 Table 3 classifies 407 of 969 children (42.0%) in the "other malocclusion only" group as victims of dental appearance-related bullying. Table 6 shows that in the same group only 25 children (2.6%) reported teasing, 13 (1.3%) unpleasant nicknames, 2 (0.2%) social exclusion and none reported physical bullying; the location counts for that group (classroom 8, playground 11, route to school 4, multiple 2) sum to 25. It is not clear how 407 children can meet the victim criterion while at most 25 endorse any of the four forms of victimisation the questionnaire enumerates.

 The same problem appears at the level of the whole sample. The location counts in the Total row of Table 6 (58 + 207 + 57 + 18) sum to 340, whereas Table 3 reports 715 victims, and the percentages in that Total row are calculated on a base of 340 rather than the stated n = 2,080. For the anterior crossbite only group the location counts sum to 287, which exceeds the 280 victims reported for that group in Table 3.

 Please state explicitly, for each panel of Table 6, what the denominator is (all children in the occlusal group, or victims only), reconcile the counts with Table 3, and correct whichever table is in error. Because the central dose-response claim rests on the victim classification, this needs to be resolved before the numerical results can be relied upon.

 2. Quasi-complete separation is acknowledged but not remedied.

 The odds ratios in Table 4 (4678.491, 95% CI 504.983-43344.549; 2160.414, 95% CI 299.538-15581.948; 497.615, 95% CI 69.700-3552.681) are artefacts of the near-empty reference cell (1 victim among 703 children with normal occlusion) rather than estimates of effect. The candid limitation paragraph added in version 3 is welcome, but a caveat is not a substitute for an appropriate estimator, and quoting these values to three decimal places invites misreading. Established remedies should be applied and reported as the primary analysis: Firth penalised-likelihood logistic regression, exact logistic regression, or a Bayesian model with a weakly informative prior. Alternatively, and probably more informative for readers, report adjusted prevalence ratios or risk differences from a log-binomial or modified Poisson model with robust variance, which behave far better in this situation. The Abstract and Discussion should then be rephrased around whichever estimates survive.

 3. The cluster design is used for the sample size but not for the analysis.

 Schools were the sampling units and a design effect of 2.5 was assumed when justifying the sample size, yet every reported inference - Pearson chi-square, Fisher-Freeman-Halton, binary logistic regression and ordinal logistic regression - assumes independent observations. Bullying is very plausibly clustered by school, since playground supervision and school climate operate at that level, so the standard errors are likely to be understated. Please re-analyse accounting for clustering (generalised estimating equations with cluster-robust standard errors, or a mixed-effects logistic model with a random intercept for school) and report how the estimates and confidence intervals change. Relatedly, the number of schools sampled is nowhere reported in the manuscript. Please give the number of clusters approached and included, the number of children per cluster, and the observed intracluster correlation for both anterior crossbite and bullying.

 4. The specification of the ordinal model (Table 5) is incomplete.

 The text states that the ordinal analysis was performed among children with anterior crossbite, but Table 5 reports estimates for all three ROMA grades (4n, 3n, 2n) with no reference category displayed. Please state the reference category explicitly, report the number of observations included in the model, and report a test of the proportional-odds assumption together with model fit statistics. If proportional odds does not hold, a partial proportional-odds or multinomial model should be substituted. More generally, since the outcome is a reverse-coded sum of four yes/no items, a binomial or count model on the number of bullying forms endorsed would be considerably easier for readers to interpret than a 4-to-8 scale on which lower values mean worse exposure.

 5. The exact p-value reported for physical bullying is not attainable.

 Table 6 records a single instance of physical bullying in the entire sample (1 child in the group of 31 with malocclusion and anterior crossbite), yet reports a Fisher-Freeman-Halton value of 67.483 with p < 0.001. With one event distributed across groups of 703, 31, 969 and 377 children, the smallest attainable exact p-value is of the order of 31/2080 = 0.015, so p < 0.001 cannot be correct. Please recheck this cell and, in any case, describe the physical-bullying comparison as uninformative rather than significant. The social exclusion comparison rests on 12 events in total and should likewise be reported with explicit counts and interpreted cautiously.

 6. Risk of information bias should be addressed directly.

 A bullying prevalence of 0.1% (1 of 703) in the normal-occlusion group is far below the 7-47.8% range for appearance-related bullying that the authors themselves cite, and the contrast with 74.3% and 87.1% in the crossbite groups is unusually sharp for self-reported psychosocial data. The manuscript does not state whether the questionnaire was administered before or after the clinical examination, whether the person administering it was blinded to the child's occlusal status, or whether the examiner (FB) was present. Children were also assessed under the teacher's supervision, which may itself influence disclosure. Please report these procedural details and discuss differential misclassification as a candidate explanation for the near-zero rate in the control group. At present the Discussion reads this figure in the opposite direction, concluding that the structural dental anomaly "is the primary target of peer aggression", which is a causal claim the design cannot support and which sits awkwardly with the stated intention to keep the framing strictly associational.

 7. Errors and inconsistent definitions in Table 1.

 In Table 1, "Absent 167 (80.4%)" should read 1,672 and "Total 208 (100.0%)" should read 2,080. In addition, three different definitions of the ROMA grades appear in the manuscript: the Methods define 2N/3N/4N by treatment urgency, Table 1 defines them by crossbite depth (4n > 2 mm, 3n > 1 mm, 2n < 1 mm), and the Results text describes 3n as malocclusion that could persist or worsen and 2n as requiring routine monitoring. The millimetre cut-offs as written also leave a gap, since a crossbite of exactly 1-2 mm falls between the stated categories. Please give one consistent operational definition with unambiguous cut-offs and use the same grade labels throughout.

 8. The comparison group is undifferentiated.

 Anterior open bite, deep bite and increased overjet were all assessed, but their individual prevalences are never reported and all 969 children are pooled as "other malocclusion only". Increased overjet in particular has the strongest prior evidence for appearance-related teasing, so pooling it with deep bite obscures the comparison that most needs to be made. Please report the distribution of each trait and, if numbers permit, disaggregate the association analysis by trait.

 9. Reporting completeness (STROBE).

 Please report the number of schools and children approached, consented, examined and excluded (with reasons), and the realised response rate - a 10-15% non-response was assumed a priori but the actual figure is never given. A participant flow diagram would settle this. The SPSS version and the specific procedures used should also be stated, together with whether any data were missing and how missingness was handled.

 MINOR POINTS

 - Typographical and copy-editing errors, including "34.4%reported" (Abstract), "and2% required" (Results), "Accounting for a potential 10-15% non-response rate of,23" (Methods), and the incomplete sentence "When precise measurements were required to support.15" (Methods).

 - The regional prevalence sentence in the Discussion is garbled: "ranging from (5.5%) in Iraq to 14% in Palestine, with Egypt and Jordan reporting 10.1% and (6.8%) respectively41,42 Jordan.43,44". Parenthesised percentages are used inconsistently and the mapping of citations to countries is unclear.

 - The claim that improved school supervision and playground monitoring reduce bullying rates carries no citation in the sentence in which it is made; presumably reference 54 is intended.

 - The observed prevalence of 19.6% is contrasted with a global estimate of about 11%, but the case definition used here includes crossbites of less than 1 mm. Part of the difference may therefore be definitional rather than epidemiological, and this deserves a sentence in the Discussion.

 - The severity score is reverse-coded (Yes = 1, No = 2; range 4-8; lower = worse). Even with the explanatory note added in version 3, consider recoding so that higher values mean more bullying, and report the observed distribution of the score.

 - References: reference 1 lists "Stomatologic, S" as an author, which appears to be a journal title captured in the author field; reference 22 ("Manual, A. P: Geneva. 1991") is incomplete; references 24 and 29 are the same Bujang et al. paper cited twice; and reference 25 gives the publisher as "John Wesley & Sons" rather than John Wiley & Sons.

 CONCLUSION

 The study addresses a question that deserves attention, and the open deposition of the dataset makes the issues above tractable. Points 1, 2, 3 and 5 are the ones I regard as necessary for the article to be scientifically sound; the remainder would improve clarity and completeness. I would be pleased to review a revised version.

Is the work clearly and accurately presented and does it cite the current literature?

Partly

If applicable, is the statistical analysis and its interpretation appropriate?

Partly

Are all the source data underlying the results available to ensure full reproducibility?

Yes

Is the study design appropriate and is the work technically sound?

Partly

Are the conclusions drawn adequately supported by the results?

Partly

Are sufficient details of methods and analysis provided to allow replication by others?

Partly

Reviewer Expertise:

Orthodontics

We confirm that we have read this submission and believe that we have an appropriate level of expertise to confirm that it is of an acceptable scientific standard, however we have significant reservations, as outlined above.

# Reviewer report: Reviewer response for version 3

**DOI:** 10.5256/f1000research.204078.r496045

**Published:** 2026-06-26

**Authors:** Tiago Fialho

**Affiliations:** 1 Bauru Dental School, São Paulo University, São Paulo, Brazil

I have completed my final review of the revised manuscript entitled "How Anterior Crossbite Severity Relates to Appearance-Based Bullying in School-Age Children: Evidence from the ROMA Index" (Version 3; DOI: 10.12688/f1000research.179073.3).

 The authors have responded thoroughly and systematically to all concerns raised in my previous review. They have appropriately acknowledged the sample size expansion as a post-hoc rationalization in the limitations section, provided a transparent discussion of the quasi-complete separation affecting the extreme odds ratios in Table 4, and reanalyzed all comparisons in Table 6 using the Fisher-Freeman-Halton exact test, which now correctly indicates that bullying locations do not differ significantly across occlusal groups. They have also explicitly noted the absence of age-by-crossbite interaction analysis as a limitation and adjusted the wording throughout to avoid any overstatement of causality, including the replacement of "enables" with "may facilitate" in the conclusion.

 The minor limitation regarding the short interval for reliability testing has also been duly acknowledged. All revisions are appropriate and have substantially improved the clarity, rigor, and transparency of the manuscript. The study addresses a clinically relevant question with a large and carefully analyzed dataset, and the findings make a meaningful contribution to the literature on malocclusion and psychosocial outcomes in children.

 I am satisfied that the manuscript now meets the standards for indexing in F1000Research. Accordingly, I recommend acceptance in its current form.

Is the work clearly and accurately presented and does it cite the current literature?

Yes

If applicable, is the statistical analysis and its interpretation appropriate?

Partly

Are all the source data underlying the results available to ensure full reproducibility?

Partly

Is the study design appropriate and is the work technically sound?

No

Are the conclusions drawn adequately supported by the results?

No

Are sufficient details of methods and analysis provided to allow replication by others?

No

Reviewer Expertise:

Orthodontics

I confirm that I have read this submission and believe that I have an appropriate level of expertise to confirm that it is of an acceptable scientific standard.

# Reviewer report: Reviewer response for version 2

**DOI:** 10.5256/f1000research.202692.r491972

**Published:** 2026-06-15

**Authors:** Tiago Fialho

**Affiliations:** 1 Bauru Dental School, São Paulo University, São Paulo, Brazil

I have completed a thorough peer review of the revised manuscript entitled "How Anterior Crossbite Severity Relates to Appearance-Based Bullying in School-Age Children: Evidence from the ROMA Index" (Version 2; DOI: 10.12688/f1000research.179073.2), originally submitted by M. Babakurd and colleagues.

 I acknowledge that this is the second version of the manuscript, following an initial round of peer review in which the first reviewer recommended against acceptance due to fundamental statistical errors, particularly concerning the binary logistic regression analysis presented in Table 4 of Version 1. Having carefully examined Version 2, I am pleased to report that the authors have systematically addressed the most critical issues raised. Nevertheless, several remaining concerns require attention before the manuscript can be considered fully suitable for indexing.

 I begin with the positive aspects of this work. The research question is clinically relevant and timely, as the relationship between malocclusion severity and psychosocial outcomes in children has gained increasing attention in orthodontic literature, yet data from Middle Eastern populations, particularly Syria, remain scarce. The authors should be commended for their large sample size of 2,080 children, which, despite some concerns regarding sample size justification discussed below, provides considerable statistical power and precision for most analyses. The use of the ROMA Index for clinical assessment represents a methodological strength, as this index offers standardized, reproducible, and internationally recognized grading of malocclusion severity, moving beyond simple binary classifications of presence or absence of crossbite. Furthermore, the modification and validation of the Olweus Bully/Victim Questionnaire for dental appearance-related bullying was conducted with appropriate rigor: the pilot study demonstrated good psychometric properties, including a Content Validity Index of 0.92, Cronbach's alpha of 0.85 for internal consistency, and an intraclass correlation coefficient of 0.88 for test-retest reliability.

 The authors also deserve praise for their commitment to transparency and reproducibility, as they have deposited their underlying data, consent forms, and questionnaires in the Open Science Framework repository (DOI: 10.17605/ OSF.IO/HW8KM), which aligns with best practices in open science. Finally, the focus on severity-based analysis rather than simply comparing presence versus absence of anterior crossbite represents a conceptual advance that enriches our understanding of the dose-response relationship between clinical conditions and social victimization.

 Turning to the critical issues that led to the initial rejection, I have carefully verified that the authors corrected the binary logistic regression error in Table 4 of Version 2. In Version 1, the odds ratios for children with malocclusion and anterior crossbite were reported as 0.000 (95% CI: 0.000 to 0.003) and for anterior crossbite only as 0.231 (95% CI: 0.176 to 0.303), which mathematically indicated a protective effect and directly contradicted the descriptive data showing 87.1% and 74.3% bullying victimization rates in those groups, respectively, compared to only 0.1% in the normal occlusion group. This error has been rectified in Version 2. The revised Table 4 now presents odds ratios that are consistent with the descriptive statistics: children with both malocclusion and anterior crossbite demonstrate an odds ratio of 4678.491 (95% CI: 504.983 to 43344.549, p < 0.001), those with anterior crossbite only show an odds ratio of 2160.414 (95% CI: 299.538 to 15581.948, p < 0.001), and those with other malocclusion only present an odds ratio of 497.615 (95% CI: 69.700 to 3552.681, p < 0.001), all compared to the normal occlusion reference group. The ordinal logistic regression analysis in Table 5, which demonstrated a dose-response relationship between ROMA severity grades and bullying intensity, remains conceptually sound and has been properly interpreted with attention to the inverse scoring of the modified Olweus scale.

 The authors have also moderated causal and speculative language throughout the manuscript, adopting a purely associational framework as recommended by the first reviewer. Specifically, they no longer claim causal inference from their cross-sectional design, and they have acknowledged that unmeasured confounders such as baseline self-esteem, pre-existing psychological vulnerability, family support structures, and socioeconomic status may influence the observed associations. These corrections are substantial and demonstrate responsiveness to peer review.

 Despite these important improvements, several methodological and reporting concerns persist and must be addressed before the manuscript can be accepted for indexing. The most significant remaining issue concerns the sample size justification. The authors initially calculated that approximately 150 children would be needed for prevalence estimation and approximately 236 children for the association between anterior crossbite and bullying, based on standard formulas for cross-sectional studies with 95% confidence level and 80% power. However, they ultimately recruited 2,080 children, nearly ten times the initial estimate. While a larger sample is not inherently problematic, the justification provided in Version 2 raises concerns about post-hoc rationalization. The authors invoke a design effect (Deff) of 2.5 due to cluster sampling, which is a reasonable adjustment, and they further argue that complex multivariable modeling, including ordinal logistic regression analyses with Mult categorical subgroups, necessitated a considerably larger sample to prevent model overfitting and ensure adequate cell frequencies. They also cite the need to detect small, expected effect sizes of f² = 0.02 with statistical power of 0.90. However, given that the observed associations are extraordinarily strong (Cramer's V = 0.578 for the main association, and odds ratios in the thousands), the justification of needing a sample size of 2,080 to detect a "small effect size" appears inconsistent. In fact, such strong associations could have been reliably detected with a much smaller sample. This discrepancy suggests that the sample size was likely determined by convenience or by the availability of schools and children rather than by a strict a priori power calculation based on expected effect sizes. While this does not invalidate the findings, it weakens the transparency of the study design and should be acknowledged as a limitation. The authors should explicitly state in the limitations section that the final sample size was larger than strictly necessary for the primary associations and that the initial sample size calculation was expanded primarily to enable stable estimation of subgroup effects and complex models, rather than for detecting the main effect.

 A second concern relates to the extraordinarily high odds ratios and their extremely wide confidence intervals in Table 4. For example, the odds ratio for the malocclusion and anterior crossbite group is 4678 with a 95% confidence interval ranging from 505 to 43344, spanning nearly two orders of magnitude. Such wide intervals indicate low precision, which is a direct consequence of the small number of children in this subgroup (n = 31, as shown in Table 3) and the near-zero prevalence of bullying victimization in the normal occlusion group (only 1 out of 703 children, or 0.1%). This pattern raises the possibility of quasi-complete separation in the logistic regression model, a phenomenon that occurs when a predictor perfectly or almost perfectly predicts the outcome in one category. In such situations, maximum likelihood estimation can produce unstable odds ratios that are inflated and have poor coverage properties. While the direction of the association is undoubtedly correct—children with anterior crossbite have vastly higher bullying rates—the precise magnitude of the odds ratio should be interpreted with caution. The authors do not discuss this issue in the limitations section, nor do they report having conducted any sensitivity analyses, such as Firth's penalized likelihood logistic regression, which is specifically designed to handle separation problems. I recommend that the authors either perform such sensitivity analyses or, at minimum, add a paragraph in the discussion acknowledging that the extreme odds ratios and wide confidence intervals reflect both the strength of the association and the statistical instability arising from the sparse data in the reference group and the small subgroup with combined malocclusion and anterior crossbite.

 Third, the description of bullying patterns and locations in Table 6, while informative, presents some interpretive challenges. The authors have now reintegrated the normal occlusion group as a baseline comparative anchor, which addresses the previous reviewer's concern. However, the statistical comparisons across four groups where one group (normal occlusion) has virtually no events (only one child reported teasing, and no child reported any other form of bullying) violate the assumption of the chi-square test that expected cell frequencies should be five or more. While the authors note that "no expected count less than five" in the footnote to Table 3, this claim is difficult to reconcile with the data in Table 6, where numerous cells have zero counts. For instance, in the analysis of unpleasant nicknames, the normal occlusion group has zero events, and the expected frequency under the null hypothesis would be substantially less than five. The p-values reported for these comparisons should therefore be interpreted with caution, as the chi-square approximation may be unreliable. The authors should consider using Fisher's exact test for these comparisons or explicitly acknowledge this limitation in the text.

 Fourth, the age effect reported in Table 4 deserves more careful discussion. The authors found that each one-year increase in age elevated the likelihood of victimization by 23.9% (OR = 1.239, 95% CI: 1.111 to 1.383, p < 0.001). This finding is interesting and potentially important, as it suggests that older children within the 8-to-12-year range are at higher risk. However, the authors do not explore whether this age effect interacts with occlusal status. For example, is the association between anterior crossbite and bullying stronger in older children than in younger ones? Such interaction analysis would be valuable and could be performed by including an age-by-crossbite interaction term in the logistic regression model. If the authors have data to conduct this analysis, I encourage them to do so. If not, they should at least acknowledge that the age effect was examined only as a covariate and that potential moderation by occlusal status remains unexplored.

 Fifth, while the authors have moderated causal language as requested, some phrasing remains overly assertive for a cross-sectional study. For example, in the abstract, they state that "anterior crossbite severity serves as a meaningful correlate" (which is acceptable) but in the conclusion they write that "early identification of anterior crossbite severity during mixed dentition enables timely recognition of children with an increased likelihood of experiencing psychosocial harm." This is acceptable as a statement of association, but the phrase "enables timely recognition" might be misread as implying that screening for crossbite will necessarily lead to reduced bullying, which is not demonstrated by this study. I suggest replacing "enables" with "may facilitate" to better reflect the exploratory nature of cross-sectional evidence.

 Sixth, although the authors have provided detailed information about the ROMA Index and its application, I note that the reliability assessment (Cohen's kappa values ranging from 0.85 to 0.95) was performed on only 10% of participants with repeat examinations spaced 20 to 30 minutes apart. This short interval may artificially inflate kappa values because memory effects could influence the second examination. Ideally, repeat examinations should be separated by at least one week to minimize recall bias. The authors should acknowledge this as a minor limitation.

 On balance, the manuscript has improved substantially from Version 1. The critical statistical error has been corrected, the language has been appropriately moderated, and the authors have demonstrated responsiveness to peer review. The research question remains important, the dataset is valuable, and the main finding—that anterior crossbite severity shows a strong, dose-response association with appearance-related bullying in Syrian schoolchildren—is both clinically relevant and methodologically supported after the corrections.

 However, the remaining issues concerning sample size justification, instability of extreme odds ratios, potential quasi-complete separation, sparse data in chi-square comparisons, and lack of interaction analysis preclude acceptance in the current form. These issues are not fatal, but they require attention.

 Therefore, my recommendation is: **Accept with minor revisions (Indexable after minor revisions)**. Specifically, I request that the authors:

 (1) add a paragraph to the limitations section acknowledging that the sample size was substantially larger than necessary for the primary associations and providing a transparent justification for the expansion;

 (2) conduct sensitivity analyses using Firth's penalized likelihood logistic regression to verify the stability of the odds ratios in Table 4, or, if such analyses are not feasible, explicitly acknowledge the potential for quasi-complete separation and the resulting imprecision;

 (3) either recalculate the p-values in Table 6 using Fisher's exact test for comparisons involving zero cells or explicitly acknowledge the limitation of chi-square approximation under sparse data;

 (4) consider testing an age-by-crossbite interaction or at least acknowledge its absence as a limitation; and

 (5) make minor wording adjustments to avoid any remaining overstatements of causality.

 These revisions are relatively modest and can be completed without new data collection or reanalysis of the entire dataset. Once these changes are implemented, I believe the manuscript will meet the standards for indexing in F1000Research.

 Respectfully submitted.

Is the work clearly and accurately presented and does it cite the current literature?

Yes

If applicable, is the statistical analysis and its interpretation appropriate?

Partly

Are all the source data underlying the results available to ensure full reproducibility?

Partly

Is the study design appropriate and is the work technically sound?

No

Are the conclusions drawn adequately supported by the results?

No

Are sufficient details of methods and analysis provided to allow replication by others?

No

Reviewer Expertise:

Orthodontics

I confirm that I have read this submission and believe that I have an appropriate level of expertise to confirm that it is of an acceptable scientific standard, however I have significant reservations, as outlined above.

# Response

**Published:** 2026-06-15

**Authors:** farah babakrd

We would like to express our sincere gratitude to the reviewer for their valuable time, comprehensive evaluation, and encouraging feedback regarding the revised version of our manuscript. We are pleased to note that the amendments made to the binary logistic regression analysis (Table 4) and the adjustments to the causal language have effectively addressed the critical issues raised during the initial review. We greatly appreciate the reviewer’s positive remarks concerning our sample size, the application of the ROMA Index, the validation of the modified Olweus Questionnaire, and our dedication to open science practices. Your constructive suggestions have significantly improved the quality and clarity of our work.

 ..............

 Reviewer's comment: 

 "The most significant remaining issue concerns the sample size justification. The authors should explicitly state in the limitations section that the final sample size was larger than strictly necessary for the primary associations and that the initial sample size calculation was expanded primarily to enable stable estimation of subgroup effects and complex models."

 Response: 

 We appreciate the reviewer's insightful observation regarding the discrepancy between our initial sample size calculation and the final sample size, particularly in light of the robust effect sizes observed. We acknowledge that the larger sample size was, in part, influenced by the feasibility and availability of the school clusters and exceeded the requirements necessary to detect the primary effects.

 In accordance with the reviewer's suggestion, we have incorporated this acknowledgment into the Limitations section. We have clarified that, although the substantial sample size was not strictly essential for detecting the primary associations, it was instrumental in ensuring statistical stability and adequate cell frequencies in our complex subgroup analyses, thus mitigating the risk of model overfitting.

 The Limitations section has been revised as follows: 

 "Another limitation pertains to the determination of the sample size. Our initial a priori power analysis indicated that a sample of approximately 236 children would suffice to identify primary associations; however, the final recruited sample consisted of 2,080 children. Although this larger sample was not strictly necessary to detect the main effects, it proved essential for ensuring the stability and adequacy of cell frequencies in complex multivariable modeling, such as the ordinal logistic regression with multiple categorical subgroups, thus preventing model overfitting. It is important to acknowledge that the final sample size reflects, in part, the opportunistic availability of the school clusters, which represents a potential post-hoc rationalization.

 …………….

 Reviewer's comment:

  "A second concern relates to the extraordinarily high odds ratios and their extremely wide confidence intervals in Table 4. This pattern raises the possibility of quasi-complete separation in the logistic regression model."

 Response:

  We sincerely appreciate the reviewer for highlighting this significant methodological concern. The reviewer correctly notes that the near-zero prevalence of bullying victimization within the normal occlusion reference group (1 out of 703) and the limited sample size of the combined malocclusion and crossbite subgroup (n=31) have led to the occurrence of quasi-complete separation. This statistical phenomenon accounts for the inflated maximum likelihood estimates and the broad confidence intervals evident in Table 4.

 In accordance with the reviewer's valuable suggestion, we have opted to explicitly acknowledge and discuss this statistical instability in the Limitations section, rather than employing complex Firth's penalized models that could obscure the presentation of the principal tables. We have provided careful guidance to readers, emphasizing that while the directional and clinical significance of the association is unequivocal (i.e., anterior crossbite is strongly correlated with elevated bullying rates), the exact numerical magnitude of the odds ratios should be interpreted with caution.

 The following paragraph has been incorporated into the Limitations section:

 "Moreover, a statistical limitation warrants caution when interpreting the precise magnitude of the odds ratios (ORs) and their wide 95% confidence intervals as presented in Table 4. This instability arises from sparse data in specific cells—namely, the near-zero prevalence of bullying victimization within the normal occlusion reference group (1 out of 703 children, representing 0.1%) in conjunction with the small sample size in the subgroup of joint malocclusion and anterior crossbite (n = 31). This distribution leads to the phenomenon of quasi-complete separation in the logistic regression model, where maximum likelihood estimation may yield inflated ORs with poor coverage properties[ 54]. Although the directional framework and the considerable strength of the association remain clinically relevant—indicating that children with anterior crossbite are at significantly elevated risk for bullying—the specific numerical values of these ORs should be regarded as indicators of a strong trend rather than precise absolute effects."

 Additionally, the following reference has been added to the reference list: 

 [54] Hosmer, D. W., Lemeshow, S., & Sturdivant, R. X. (2013). Applied Logistic Regression (3rd ed.). John Wiley & Sons.

 ………….

 Reviewer’s Comment: 

 "Third, the description of bullying patterns and locations in Table 6 violates the assumption of the chi-square test that expected cell frequencies should be five or more. The authors should consider utilizing Fisher's exact test for these comparisons or explicitly acknowledge this limitation in the text."

 Response: 

 We express our sincere gratitude for the reviewer’s insightful and constructive methodological recommendation. We concur that the occurrence of multiple zero and low counts in Table 6 contravenes the standard assumption of the chi-square test, thereby rendering the asymptotic p-values less reliable.

 Instead of merely acknowledging this as a limitation, we have taken the initiative to re-analyze all comparisons presented in Table 6 using the Fisher-Freeman-Halton exact test, which is the appropriate exact probability test for R × C contingency tables, to ensure absolute statistical rigor.

 Consequently, Table 6 has been comprehensively updated to include the exact p-values. It is noteworthy that the exact p-values for all bullying patterns, namely Teasing, Nicknames, Social Exclusion, and Physical Bullying, remained highly significant (p < 0.001). However, the exact test indicated that the differences in bullying locations across the groups do not reach statistical significance (p = 0.120), corrected from the previous unstable asymptotic (p = 0.043). We have revised our descriptive interpretations in the Results and Discussion sections to accurately reflect these statistically robust findings.

 ………........

 Reviewer’s Comment: 

 "Fourth, the age effect reported in Table 4 warrants more thorough discussion. However, the authors do not explore whether this age effect interacts with occlusal status. If the authors possess data to conduct this analysis, I encourage them to do so. If this is not the case, they should at least acknowledge that the age effect was examined solely as a covariate and that potential moderation by occlusal status remains unexamined."

 Response: 

 We express our gratitude to the reviewer for underscoring the significant observation regarding the age effect and for proposing an analysis of potential interaction between age and occlusal status. The reviewer is correct that investigating whether age functions as a moderator could yield valuable insights into the developmental dynamics associated with appearance-based bullying.

 In this study, age was conceptually introduced and utilized strictly as a continuous covariate to account for confounding variables and to isolate the independent impact of occlusal status on bullying victimization. In light of the reviewer’s insightful suggestion, we have chosen to explicitly acknowledge that potential moderation or interaction effects between age and malocclusion severity remain unexplored within our current model. We have incorporated this acknowledgment into the revised Limitations section, emphasizing it as an important avenue for future longitudinal or targeted psychological research.

 The following text has been added to the Limitations section: 

 "Additionally, although chronological age was adjusted for as a significant covariate within the multivariable logistic regression model—indicating an increased likelihood of bullying victimization with age—potential interaction effects between age and occlusal status (i.e., whether age moderates the impact of anterior crossbite severity on bullying) were not explicitly modeled. This oversight leaves the potential moderating role of developmental stage unexamined, which represents an area for future inquiry."

 ..........

 Reviewer's comment:

 "Fifth, while the authors have moderated causal language as requested, some phrasing remains overly assertive... I suggest replacing 'enables' with 'may facilitate' to better reflect the exploratory nature of cross-sectional evidence."

 Response:

 We sincerely thank the reviewer for this precise and helpful textual suggestion. We agree that the word "enables" may sound overly deterministic and could inadvertently imply a proven clinical outcome beyond the scope of a cross-sectional design. Following your excellent recommendation, we have replaced "enables" with "may facilitate"

 ....

 Reviewer's Comment: 

 "Sixth, while the authors have provided comprehensive information regarding the ROMA Index and its application, I observe that the reliability assessment was conducted on only 10% of participants, utilizing repeat examinations spaced 20 to 30 minutes apart. This brief interval may artificially inflate kappa values due to potential memory effects influencing the second examination. The authors should acknowledge this as a minor limitation."

 Response: 

 We fully acknowledge the reviewer's insightful observation concerning the possible influence of memory effects on the repeat examinations. The reviewer is correct in noting that the 20-to-30-minute interval is relatively brief, which may introduce recall bias and subsequently inflate the Cohen's kappa values.

 In response, we have explicitly recognized this as a minor limitation within our revised manuscript. We have clarified that a longer interval—such as one week—would have been methodologically preferable to mitigate the effects of recall bias; however, the limited washout period was necessitated by stringent school scheduling and logistical considerations during the field study.

 The following text has been incorporated into the updated Limitations section : 

 "Lastly, a minor methodological limitation pertains to the reliability assessment of the ROMA Index. The repeat examinations for a 10% sub-sample were conducted 20 to 30 minutes apart; this brief interval may have introduced memory or recall bias, potentially inflating the Cohen's kappa values (ranging from 0.85 to 0.95). While a longer washout period, such as one week, would have been preferable to minimize memory effects completely, logistical constraints within the school settings necessitated this immediate re-examination protocol. Consequently, future longitudinal, multicenter studies should be undertaken to elucidate temporal pathways, evaluate the long-term psychosocial benefits of early orthodontic interventions, and assess the cost-effectiveness of incorporating malocclusion screening into standard school health programs.

 ………………..

 We are extremely pleased with the reviewer’s favorable final evaluation and the recommendation for indexing, contingent upon minor revisions. We sincerely appreciate your detailed guidance throughout the peer-review process, which has significantly enhanced the statistical rigor and academic quality of our manuscript. Thank you once again for your invaluable expertise. We are confident that the manuscript is now thoroughly optimized and ready for indexing in F1000Research.

# Reviewer report: Reviewer response for version 1

**DOI:** 10.5256/f1000research.197541.r485889

**Published:** 2026-05-26

**Authors:** Tiago Fialho

**Affiliations:** 1 Bauru Dental School, São Paulo University, São Paulo, Brazil

Thank you for the opportunity to review the manuscript entitled “How Anterior Crossbite Severity Relates to Appearance-Based Bullying in School-Age Children: Evidence from the ROMA Index” (version 1; DOI: 10.12688/f1000research.179073.1). I have completed a thorough critical appraisal of this work. While the research question is clinically relevant and the authors have attempted to address an important gap in the literature concerning anterior crossbite severity and psychosocial outcomes in Syrian schoolchildren, I regret to conclude that the manuscript is not acceptable for indexing in its current form due to fundamental statistical errors that compromise the validity and interpretability of the main findings.

 The most serious flaw concerns the binary logistic regression analysis presented in Table 4. The authors report odds ratios for bullying victimization comparing different occlusal status groups to the reference category of normal occlusion. According to the table, children with both malocclusion and anterior crossbite have an Exp(B) of 0.000 with a 95% confidence interval of 0.000 to 0.003, and children with anterior crossbite only have an Exp(B) of 0.231 with a 95% confidence interval of 0.176 to 0.303. Both odds ratios are substantially below 1, which mathematically indicates a strong protective effect, meaning these groups would have a much lower risk of bullying compared to children with normal occlusion. However, this directly contradicts the descriptive data presented earlier in Table 3, where 87.1% of children in the malocclusion and anterior crossbite group and 74.3% of those with anterior crossbite only were classified as bullying victims, whereas only 0.1% of children with normal occlusion were victims. The odds ratios as reported are therefore impossible to reconcile with the observed proportions, indicating a critical error in either the coding of the outcome variable, the specification of the reference category, the direction of the calculated odds ratios, or the interpretation of the regression output. Until this error is corrected and the analysis is rerun with proper verification, no reliable conclusions can be drawn from the multivariable analysis, and consequently the paper’s central claim that anterior crossbite increases bullying risk lacks valid statistical support.

 Beyond this critical error, there are additional methodological concerns that require attention. The sample size justification is problematic. The authors initially calculate that approximately 150 children would be needed for prevalence estimation and approximately 236 children for the association between crossbite and bullying, yet they ultimately recruit 2,080 children without providing a clear, statistically grounded rationale for this tenfold expansion. While a larger sample is not inherently problematic, the absence of a priori justification based on expected effect sizes, design effects from cluster sampling, or required power for subgroup and ordinal regression analyses weakens the transparency of the study design. Furthermore, the manuscript claims a dose-response relationship between crossbite severity and bullying severity based on ordinal logistic regression (Table 5), which is conceptually sound, but this finding is undermined by the unresolved inconsistency in the binary regression results. Readers and reviewers cannot fully trust the ordinal model when the more basic binary model contains an uncorrected error.

 Additionally, the authors invoke Bradford Hill criteria for causality, particularly the dose-response principle, yet the cross-sectional design of the study precludes any causal inference. The observed associations, even if correctly analyzed, could be explained by unmeasured confounders such as baseline self-esteem, pre-existing psychological vulnerability, family support structures, or socioeconomic status, none of which were included in the regression models. The language throughout the discussion, including phrases such as “causal inference” and “risk indicator” being interpreted as more than associative, overreaches the limitations of the study design. The authors should reframe their conclusions to reflect only associational findings.

 There are also several reporting inconsistencies that detract from the manuscript’s clarity. On page 4, the text refers to “anterior cross-profile prevalence” and “anterior cross-site” instead of anterior crossbite, suggesting typographical errors that should be corrected. The description of the ROMA grading system indicates that higher grades represent greater severity, yet the interpretation of the ordinal regression coefficients requires careful attention to the coding of bullying severity scores, where lower scores paradoxically indicate greater bullying exposure. While the authors acknowledge this coding scheme, the presentation remains confusing for readers not intimately familiar with the modified Olweus scoring method. Finally, the exclusion of children with normal occlusion from the analysis of bullying patterns and locations (Table 6) is justified on the grounds that bullying was negligible in this group, but this decision removes an important comparative anchor and limits the interpretability of the location data.

 On a positive note, the study has several strengths that should be acknowledged. The use of the ROMA Index provides a standardized and reproducible clinical assessment, and the pilot testing of the modified Olweus questionnaire demonstrated good psychometric properties with a content validity index of 0.92, Cronbach’s alpha of 0.85, and an intraclass correlation coefficient of 0.88. The sample size of 2,080 children is impressive and, if properly analyzed, could provide precise estimates. The authors should also be commended for making their data and materials available through the Open Science Framework, which aligns with F1000Research’s commitment to transparency and reproducibility. The study addresses a genuinely under-researched population, namely children in Syria, and the focus on severity-based analysis rather than simple presence or absence of malocclusion is conceptually valuable.

 I believe the research question remains important and the dataset has potential value. Therefore, I encourage the authors to thoroughly revise the statistical analysis, specifically correcting the binary logistic regression error and ensuring consistency across all tables and interpretations. They should also provide a clearer and more justified sample size calculation, tone down causal language, and improve reporting consistency. Once these revisions are made, the manuscript could be resubmitted as a new version for further review. Given the severity of the statistical error, I cannot recommend acceptance at this stage, but I remain open to evaluating a corrected version in the future.

Is the work clearly and accurately presented and does it cite the current literature?

Yes

If applicable, is the statistical analysis and its interpretation appropriate?

Partly

Are all the source data underlying the results available to ensure full reproducibility?

Partly

Is the study design appropriate and is the work technically sound?

No

Are the conclusions drawn adequately supported by the results?

No

Are sufficient details of methods and analysis provided to allow replication by others?

No

Reviewer Expertise:

Orthodontics

I confirm that I have read this submission and believe that I have an appropriate level of expertise to state that I do not consider it to be of an acceptable scientific standard, for reasons outlined above.
